# Integrative Multi-Omics and Single-Cell Profiling Identify Chitinase Domain Containing Protein 1 (CHID1) as a Prognostic Biomarker in Glioblastoma

**DOI:** 10.7150/jca.130519

**Published:** 2026-03-17

**Authors:** Sachin Kumar, Chung-Che Wu, Dahlak Daniel Solomon, Juan Lorell Ngadio, Do Thi Minh Xuan, Ching-Chung Ko, Neethu Palekkode, Ayman Fathima, Hung-Yun Lin, Hui-Ru Lin, Chih-Yang Wang, Yung-Kuo Lee, Ngoc Uyen Nhi Nguyen

**Affiliations:** 1 Ph.D. Program for Cancer Molecular Biology and Drug Discovery, College of Medical Science and Technology, Taipei Medical University, Taipei 11031, Taiwan.; 2 Graduate Institute of Cancer Biology and Drug Discovery, College of Medical Science and Technology, Taipei Medical University, Taipei 11031, Taiwan.; 3 Faculty of Applied Sciences and Biotechnology, Shoolini University of Biotechnology and Management Sciences, Himachal Pradesh, 173229, India.; 4 Department of Neurosurgery, Taipei Medical University Hospital, Taipei, Taiwan.; 5 Department of Surgery, School of Medicine, College of Medicine, Taipei Medical University, Taipei, Taiwan.; 6 Yogananda School of AI Computers and Data Sciences, Shoolini University, Solan 173229, India.; 7 Department of Bioinformatics, School of Life Sciences, Indonesia International Institute for Life Sciences, Jl Pulomas Barat Kav 88, Jakarta Timur 13210, Indonesia.; 8 Faculty of Pharmacy, Van Lang University, 69/68 Dang Thuy Tram Street, Binh Tri Dong Ward, Ho Chi Minh City 70000, Vietnam.; 9 Department of Medical Imaging, Chi-Mei Medical Center, Tainan City 71004, Taiwan.; 10 Department of Health and Nutrition, Chia Nan University of Pharmacy and Science, Tainan City 71710, Taiwan.; 11 School of Medicine, College of Medicine, National Sun Yat-Sen University, Kaohsiung 80424, Taiwan.; 12 Department of Biotechnology, Mother Teresa Women's University, Kodaikanal, Tamil Nadu, 624101, India.; 13 School of Computer Science and Engineering, Presidency University, Yelahanka, Bengaluru 560064 India.; 14 Traditional Herbal Medicine Research Center of Taipei Medical University Hospital, Taipei Medical University, Taipei 11031, Taiwan.; 15 Pharmaceutical Research Institute, Albany College of Pharmacy and Health Sciences, Rensselaer, NY 12144, USA.; 16 Cancer Center, Wan Fang Hospital, Taipei Medical University, Taipei 11031, Taiwan.; 17 TMU Research Center of Cancer Translational Medicine, Taipei Medical University, Taipei 11031, Taiwan.; 18 Institute of Medical Science and Technology, National Sun Yat-Sen University, Kaohsiung 80424, Taiwan.; 19 Nursing Department, Kaohsiung Armed Forces General Hospital, National Defense Medical University, Kaohsiung 80284, Taiwan.; 20 Department of Emergency Medicine, Kaohsiung Armed Forces General Hospital, National Defense Medical University, Kaohsiung 80284, Taiwan.; 21 Medical Laboratory, Medical Education and Research Center, Kaohsiung Armed Forces General Hospital, National Defense Medical University, Kaohsiung 80284, Taiwan.; 22 Division of Experimental Surgery Center, Department of Surgery, Tri-Service General Hospital, National Defense Medical University, Taipei 11490, Taiwan.; 23 School of Medicine, National Defense Medical University, Taipei, 11490, Taiwan.; 24 Center for Regenerative Medicine, University of South Florida Health Heart Institute, Tampa, FL 33602, U.S.A.; 25 Division of Cardiology, Department of Internal Medicine, Morsani School of Medicine, University of South Florida, Tampa, FL 33602, U.S.A.

**Keywords:** Glioblastoma, GBM, CHID1, Chitinase-like proteins, Immunometabolism, Multi-omics, Single-cell RNA sequencing, Prognostic biomarker

## Abstract

Glioblastoma multiforme (GBM), the most aggressive primary brain tumor, is characterized by high recurrence, metabolic plasticity, and complex tumor microenvironmental interactions. The human chitinase and chitinase-like protein family includes five members (CHI3L1, CHI3L2, CHIA, CHID1, and CHIT1) that share conserved chitinase-related domains but exhibit diverse biological functions in immune regulation and tissue remodeling. While chitinase-like proteins are recognized as mesenchymal-associated markers, however, the role of CHID1 in GBM remains largely unexplored. An integrative multi-omics strategy combining TCGA-GBM and CGGA transcriptomic datasets, single-cell RNA sequencing, and enrichment analyses (GSEA, GO, KEGG, and MetaCore) were used to investigate CHID1 expression patterns and associated transcriptional programs. Pharmacogenomic correlations and molecular docking were used to explore potential drug-response associations. CHID1 showed higher expression in GBM compared to the normal brain and was associated with poor overall survival. A single-cell analysis showed tumor-associated expression patterns of CHID1 across malignant samples. Pathway enrichment analyses identified transcriptional programs related to oxidative phosphorylation, redox-related processes, DNA repair, and cell cycle pathways. Collectively, this study provides a comprehensive multi-cohort and multi-modal characterization of CHID1 expression in GBM, integrating bulk transcriptomics, single-cell RNA sequencing, and tissue-level validation. The findings establish CHID1 as a GBM-associated transcriptional marker linked to metabolic and redox-related programs and provide a systematic resource for future investigations into chitinase family-related biology in GBM.

## 1. Introduction

Glioblastoma multiforme (GBM) remains the most aggressive primary malignant brain tumor in adults, with a median overall survival of approximately 12-15 months despite maximal treatment consisting of surgical resection, radiotherapy, and temozolomide (TMZ) chemotherapy 1-4. The unfavorable prognosis reflects the highly infiltrative growth pattern of GBM as well as marked molecular heterogeneity, genomic instability, and adaptive transcriptional programs. These characteristics are thought to contribute to therapeutic resistance and tumor persistence within the brain microenvironment 5. Accordingly, identifying molecular correlates associated with these features remains an important objective in neuro-oncology research.

The tumor microenvironment (TME) has been increasingly implicated in GBM progression and treatment response 6, 7. Tumor-associated macrophages (TAMs), including resident microglia and infiltrating monocyte-derived macrophages, can constitute a substantial fraction of the tumor mass and frequently exhibit immunoregulatory phenotypes associated with angiogenesis, extracellular matrix remodeling, and immune suppression 8, 9. Among molecules implicated in macrophage-tumor interactions, chitinase-like proteins (CLPs) have attracted attention. In particular, CHI3L1 (YKL-40) is strongly associated with the mesenchymal GBM subtype and has been correlated with extracellular matrix remodeling, inflammatory signaling, and adverse clinical outcomes 10, 11. Elevated CHI3L1 expression has also been reported in tumors with increased macrophage infiltration, supporting a potential link between CLPs and the immune microenvironment in GBM.

In contrast to CHI3L1, chitinase domain-containing protein 1 (CHID1) remains relatively less characterized in glioma biology. Although structurally related to other CLPs, CHID1 lacks catalytic chitinase activity due to alterations within the conserved active-site residues, suggesting that its biological functions may differ from enzymatically active family members. CHID1 expression has been reported in immune-related cell types, but its relevance in GBM has not been well defined. While emerging transcriptomic studies have suggested possible associations between CHID1 expression and metabolic pathways, including oxidative phosphorylation and mitochondrial-related gene signatures, direct functional evidence remains limited. Therefore, whether CHID1 expression reflects specific metabolic or immune-related states within GBM requires further investigation 12. Therefore, whether CHID1 expression reflects specific metabolic or immune-related states within GBM requires further investigation.

Therapeutic advances in GBM have been constrained in part by the blood-brain barrier, which restricts the effective delivery of many systemic agents to the central nervous system 13, 14. Although TMZ remains the standard chemotherapeutic agent, resistance frequently develops, and many targeted therapies have shown limited clinical benefit. These challenges underscore the need for improved molecular stratification strategies and for biomarkers that may refine biological classification and guide hypothesis-driven therapeutic development.

**Figure 1:**
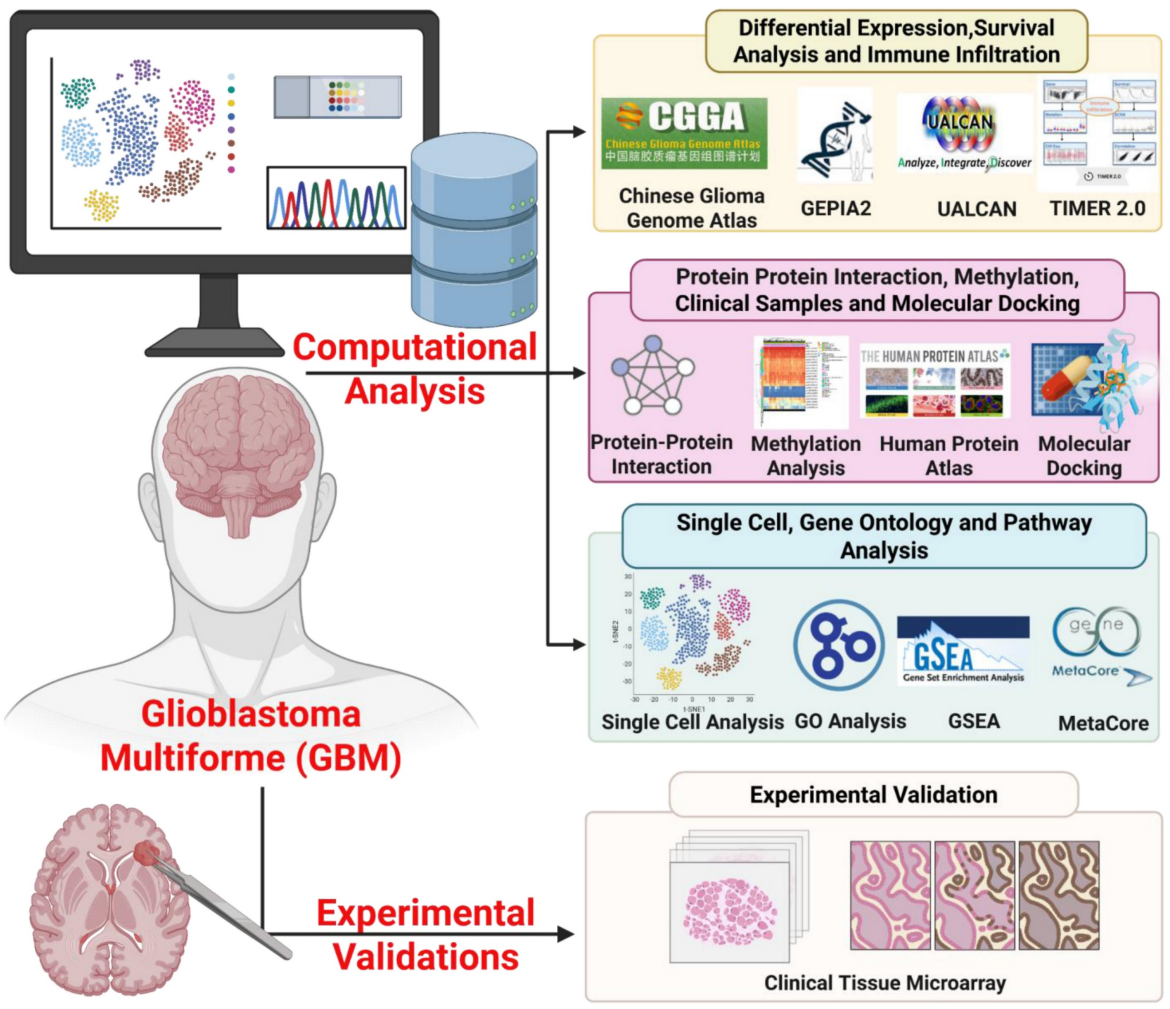
**Overview of the integrative workflow used to investigate CHID1 in GBM**. This schematic illustrates the overall study design integrating computational multi-omics analyses with experimental validation to characterize the role of CHID1 in GBM. Public transcriptomic datasets from TCGA-GBM and CGGA were analyzed to evaluate CHID1 differential expression, prognostic significance, and immune infiltration using platforms including GEPIA2, UALCAN, and TIMER2.0. Protein-protein interaction networks, DNA methylation patterns, and functional enrichment analyses were examined through STRING, MethSurv, Gene Ontology (GO), KEGG pathway analysis, GSEA, and MetaCore. Pharmacogenomic associations and molecular docking analyses were further performed to explore potential therapeutic relevance. Finally, protein-level validation of CHID1 expression was conducted using immunohistochemical staining of clinical brain tumor tissue microarrays.

In this study, we evaluated CHID1 as a candidate biomarker in GBM by integrating bulk transcriptomic datasets from The Cancer Genome Atlas (TCGA-GBM) and Chinese Glioma Genome Atlas (CGGA) with survival analyses and pathway enrichment approaches, including gene set enrichment analysis (GSEA), gene ontology (GO), Kyoto Encyclopedia of Genes and Genomes (KEGG), and MetaCore analyses. Single-cell RNA sequencing data were further examined to assess cell-type-specific expression patterns. Independent validation was performed using external transcriptomic datasets and immunohistochemical analysis of patient-derived tissue microarrays to evaluate protein-level expression. In addition, exploratory analyses were conducted to assess correlations with drug sensitivity datasets and to model potential molecular interactions through *in silico* docking. The overall analytical workflow is summarized in Figure 1. These integrative analyses were designed to clarify the transcriptional context and potential clinical associations of CHID1 expression in GBM.

## 2. Material and Methods

### 2.1 Data Collection, Expression, and Survival Analyses

Transcriptomic expression profiles and clinical data were accessed from multiple publicly available cancer genomics resources to evaluate the expression patterns and prognostic relevance of CHID1 in GBM and across pan-cancer cohorts. RNA-seq expression and survival data were analyzed using the GEPIA2 platform (http://gepia2.cancer-pku.cn/), which integrates processed expression data derived from The Cancer Genome Atlas (TCGA) and the GTEx 15. CHID1 mRNA expression levels were compared between tumor and normal tissues across 33 cancer types, and GBM-specific analyses were conducted to evaluate differential expression. 16-18. To assess the prognostic significance of CHID1 and related chitinase-like genes, survival analyses were performed using the UALCAN platform (http://ualcan.path.uab.edu), which provides survival analyses based on TCGA clinical datasets 19. Kaplan-Meier survival curves were generated to compare overall survival (OS) between high- and low-expression groups using the median expression value as the cutoff. Statistical significance was evaluated using the log-rank test, and hazard ratios (HRs) with 95% confidence intervals (CIs) were reported when available. For independent validation and subgroup analysis, expression profiles were examined using the Chinese Glioma Genome Atlas (CGGA; https://www.cgga.org.cn/) 20. Boxplots were generated to compare CHID1 expression across demographic and clinical categories, including age, sex, WHO tumor grade, and tumor progression status.

### 2.2 DNA Methylation, Protein-Protein Interaction Network, and Subcellular Localization of CHID1

To explore potential epigenetic regulation of CHID1 in GBM, DNA methylation data were analyzed using the MethSurv (https://biit.cs.ut.ee/methsurv/), which provides single-CpG resolution methylation profiles derived from the Illumina HumanMethylation450 array across TCGA tumor samples 21. Within the TCGA-GBM cohort, CpG site-specific methylation β-values associated with CHID1 were examined to visualize methylation patterns across tumor samples. Spearman correlation analyses were performed to evaluate associations between individual CpG methylation levels and CHID1 mRNA expression. The subcellular localization of CHID1 protein was assessed using data from the Human Protein Atlas (HPA; https://www.proteinatlas.org) 22. Immunofluorescence images from U-251 MG cells stained with the HPA039374 antibody were examined. Co-staining markers for nuclei (DAPI), microtubules, and endoplasmic reticulum structures were used to evaluate subcellular distribution patterns 23. To identify potential functional associations, a protein-protein interaction (PPI) network centered on CHID1 was constructed using the STRING (https://string-db.org) 24. This analysis generated confidence-scored interaction networks based on experimentally validated interactions, computational predictions, and curated database information.

### 2.3 Pathway and Functional Enrichment Analyses of CHID1

Transcriptomic enrichment analyses were conducted using several complementary computational approaches. Gene set enrichment analysis (GSEA) was performed using the fgsea package (v1.28.0) in R (v4.3.2) with transcriptomic data derived from the TCGA-GBM cohort 25, 26. Genes were ranked according to differential expression statistics between CHID1 high- and low-expression groups (median cutoff), and enrichment was evaluated using Hallmark gene sets from the MSigDB (v7.5) 30. Enrichment scores were calculated using 1000 permutations, and pathways with a false discovery rate (FDR) q value < 0.05 were considered statistically significant. To complement GSEA results, Gene Ontology (GO) and Kyoto Encyclopedia of Genes and Genomes (KEGG) enrichment analyses were conducted using the clusterProfiler (v4.10.0) 31, 32. Genes significantly co-expressed with CHID1 were obtained from the cBioPortal (https://www.cbioportal.org/) and used as input for enrichment analysis 33-35. GO analysis was performed across biological process (BP), molecular function (MF), and cellular component (CC) categories, whereas KEGG analysis was used to identify associated signaling and metabolic pathways. Statistical significance was defined as FDR q < 0.05. Results were visualized using the enrichplot and ComplexHeatmap packages. For additional pathway interpretation, MetaCore pathway analysis was performed using curated pathway databases within the MetaCore (Clarivate Analytics) 36-39. Genes showing the strongest correlations with CHID1 expression were extracted from the TCGA-GBM dataset and uploaded to the MetaCore portal for enrichment analysis using hypergeometric testing. Pathways with p < 0.05 were considered significantly enriched.

### 2.4 Immune Infiltration and Single-Cell RNA Profiling of CHID1

To evaluate the association between CHID1 expression and immune cell infiltration in GBM, we used the TIMER2.0 platform (http://timer.cistrome.org/) 40. CHID1 mRNA expression was correlated with estimated infiltration levels of major immune cell populations, including T cells, B cells, neutrophils, dendritic cells, and macrophages. Correlations were assessed using Spearman correlation coefficients. To further examine CHID1 expression at single-cell resolution, single-cell RNA sequencing data were obtained from the Gene Expression Omnibus under accession number GSE182109, which contains transcriptomic profiles from ten GBM tumor samples 41. Raw count matrices were processed using the Seurat package (v5.1.0) in R 42. Quality control steps included removal of cells expressing fewer than 200 detected genes and cells with high mitochondrial gene expression (>10-15%). After normalization and scaling, highly variable genes were identified and principal component analysis (PCA) was performed for dimensionality reduction. Cell clusters were identified using a graph-based clustering algorithm and visualized using Uniform Manifold Approximation and Projection (UMAP). Cell-type annotation was performed using canonical lineage markers and automated classification with the Single Cell Pipeline (SCP; v0.5.6). Major cell populations, including malignant glioma cells, macrophages/microglia, T cells, endothelial cells, and oligodendrocyte precursor cells, were identified. CHID1 expression was then visualized across cell populations using feature plots and heatmaps generated with the ComplexHeatmap package 43. This integrated approach combining a bulk immune infiltration analysis (TIMER2.0) with single-cell profiling (Seurat/SCP) allowed us to map CHID1 expression from the tissue-wide immune context down to the single-cell level 44-46.

### 2.5 Drug Sensitivity Correlation and Molecular Docking Analysis

To explore potential therapeutic associations with CHID1 expression, drug sensitivity correlations were evaluated using the GSCA (http://bioinfo.life.hust.edu.cn/GSCA/) 47-49. This platform integrates transcriptomic data with large-scale pharmacogenomic datasets, including the Genomics of Drug Sensitivity in Cancer (GDSC) and the Cancer Therapeutics Response Portal (CTRP). Correlations between CHID1 expression levels and drug response metrics were assessed to identify compounds whose predicted sensitivity was associated with CHID1 expression 50. To further investigate potential molecular interactions, molecular docking analyses were performed. Three-dimensional ligand structures of candidate compounds were obtained from the PubChem in SDF format and converted to PDBQT format using PyMOL and AutoDockTools 51-53. The predicted three-dimensional structure of human CHID1 was retrieved from the AlphaFold Protein Structure Database using UniProt accession Q9BWS9 24. Structural regions with low confidence scores (pLDDT < 50) were excluded from docking preparation. Potential ligand-binding pockets were predicted using the CastP-Fold server, and docking simulations were conducted using AutoDock Vina with an exhaustiveness parameter of 8 and energy range of 4. Docking results were ranked based on predicted binding affinity scores. Protein-ligand interactions, including hydrogen bonds and hydrophobic contacts, were visualized using BIOVIA Discovery Studio Visualizer (Dassault Systèmes, https://discover.3ds.com/discovery-studio-visualizer-download) 54.

### 2.6 Clinical Tissue Microarray and Immunohistochemistry

To validate CHID1 protein expression in clinical specimens, immunohistochemical staining was performed on formalin-fixed paraffin-embedded human brain tissue microarrays (TMAs; GL208a). The cohort consisted of 51 brain tumor tissues and 21 normal cerebrum tissues obtained from tissue array (KAFGH-IRB: 111-041). Representative tumor and normal regions were identified from hematoxylin and eosin-stained sections by a certified pathologist, and cylindrical tissue cores (1.5 mm diameter) were assembled into recipient paraffin blocks. Tissue sections (4 μm) were deparaffinized, rehydrated, and subjected to antigen retrieval in 10 mM sodium citrate buffer (pH 6.0). Endogenous peroxidase activity was blocked with 3% hydrogen peroxide. Slides were incubated overnight at 4 °C with a rabbit polyclonal anti-CHID1 antibody (AbClonal, cat. no. A16142) at a dilution of 1:200. After washing, sections were incubated with a biotinylated goat anti-rabbit IgG secondary antibody followed by visualization using the Vectastain ABC detection system with 3,3'-diaminobenzidine (DAB). Nuclei were counterstained with hematoxylin. Whole-slide images were analyzed using QuPath software (v0.3.2). Staining intensity was graded on a four-point scale: 0 (no staining), 1 (weak), 2 (moderate), and 3 (strong) 55-57.

### 2.7 Statistical Analysis

Bioinformatics analyses were performed using the online platforms described above. Statistical analyses and visualization were conducted using R (ggplot2 package), SPSS (IBM, Armonk, NY, USA), and ImageJ. Continuous variables are presented as mean ± standard deviation where applicable 58-62. Comparisons between two groups were performed using Student's t-test. Survival analyses were conducted using the Kaplan-Meier method, and statistical significance was evaluated using the log-rank test 63-65. A p value < 0.05 was considered statistically significant 66-70.

## 3. Results

### 3.1 Genomic Features and Differential Expressions of CHID1 and Related Chitinase-Like Genes

**Figure 2:**
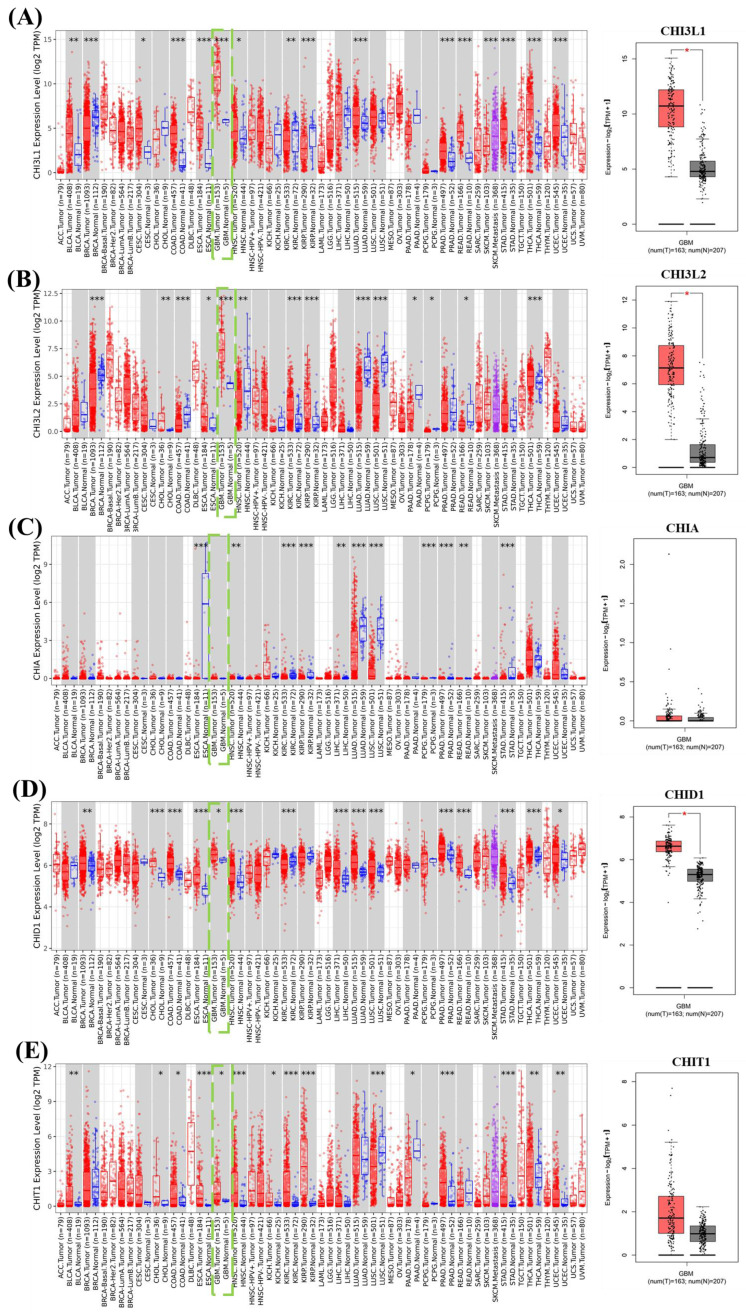
**Pan-cancer expression profiles of CHI3L family genes with focus on GBM.** Box-and-scatter plots show the expression levels of (A) CHI3L1, (B) CHI3L2, (C) CHIA, (D) CHID1, and (E) CHIT1 across multiple cancer types using integrated TCGA and GTEx datasets. Expression values are presented as log2(TPM+1). Red dots represent tumor samples and blue dots represent normal tissues. GBM samples are highlighted within the pan-cancer comparison. The right panels display direct comparisons between GBM tumor tissues and normal brain samples. Statistical significance between tumor and normal tissues is indicated by asterisks (*p < 0.05, **p < 0.01, ***p < 0.001). Sample numbers for tumor (T) and normal (N) groups are indicated below each plot*.*

CLPs belong to the glycoside hydrolase family 18 and include both enzymatically active and inactive members that are commonly associated with immune responses and tissue remodeling. Within this family, CHIT1 retains chitinase activity, whereas CHI3L1, CHI3L2, and CHID1 lack catalytic activity despite containing conserved chitin-binding motifs. These inactive CLPs are thought to function primarily through carbohydrate recognition and protein-protein interactions. Genomic annotation indicated that CHI3L1, CHI3L2, and CHIT1 are located on chromosome 1 (1q32.1 and 1p13.2), consistent with a shared evolutionary origin, whereas CHID1 is located on chromosome 11p15.5, indicating genomic separation from the chromosome 1 CLP cluster (Table 1). To characterize expression patterns, transcriptomic data from pan-cancer cohorts and GBM samples from The Cancer Genome Atlas were analyzed (Figure 2). CHI3L1 and CHI3L2 showed relatively high expression across several tumor types, with CHI3L1 displaying particularly elevated expression in GBM compared with normal brain tissues. In contrast, CHID1 showed a modest but consistent increase in GBM samples relative to normal controls, whereas CHIT1 exhibited variable expression without a consistent tumor-specific trend. Analysis of the TCGA-GBM cohort similarly demonstrated elevated CHID1 expression in GBM compared with normal brain tissues (Figure 2A-E). To validate these findings in an independent dataset, we analyzed GSE7696, which includes normal brain tissues, primary GBM, and recurrent GBM samples with treatment-stratified subgroups (radiotherapy, temozolomide plus radiotherapy, and recurrent treated tumors). Consistent with the TCGA results, CHID1 expression was moderately increased in primary GBM relative to normal tissues and was further elevated in recurrent tumors (Supplementary Figure S1A). Stratification by therapeutic exposure showed that CHID1 expression remained significantly higher in GBM samples following radiotherapy or combined temozolomide/radiotherapy compared with normal brain tissues, with recurrent treated tumors displaying the highest expression levels (Supplementary Figure S1B). Overall, these analyses indicate distinct expression patterns among chitinase-like family members in GBM. Whereas CHI3L1 showed prominent expression in mesenchymal-associated tumor contexts, CHID1 displayed reproducible tumor-associated enrichment across multiple independent datasets, supporting further investigation of its potential relevance in GBM biology.

### 3.2 Prognostic Value of CHID1 and Related Chitinase-Like Genes in GBM

**Figure 3:**
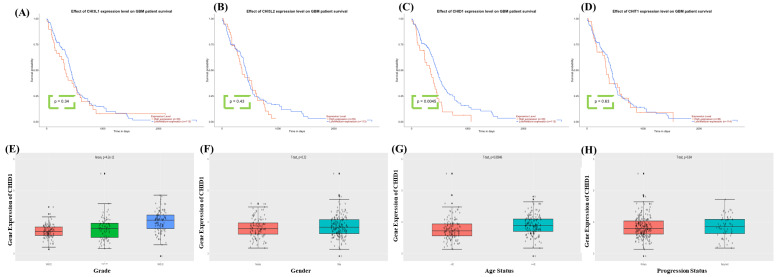
** Survival associations of chitinase-like family genes and *CHID1* in GBM.** Kaplan-Meier survival analyses showing the association between expression levels of CHI3L1, CHI3L2, CHID1, and CHIT1 and overall survival in patients with GBM (A-D). Patients were stratified into high- and low-expression groups, and survival differences were evaluated using the log-rank test. Among these genes, CHID1 expression showed a significant association with patient survival. (E-H) Box plots showing the relationship between CHID1 expression and clinicopathological characteristics in GBM, including tumor grade, gender, age status, and progression status. Gene expression levels are displayed with individual sample points, and statistical comparisons were performed using appropriate parametric tests.

To evaluate the prognostic significance of CLP family members in GBM, survival data from the TCGA-GBM cohort were analyzed. Kaplan-Meier survival analysis showed that higher CHID1 expression was significantly associated withshorter overall survival (p = 0.0045). In contrast, CHI3L1 (p = 0.34), CHI3L2 (p = 0.43), and CHIT1 (p = 0.63) showed no statistically significant association with patient survival (Figure 3A-D). These results suggest that, among the CLP family members examined, CHID1 demonstrates the strongest association with overall survival in GBM. To further examine these findings and explore potential clinical associations, CHID1 expression was evaluated using data from the Chinese Glioma Genome Atlas. CHID1 expression was significantly higher in high-grade gliomas compared with lower-grade tumors (p < 0.001) (Figure 3E). No significant differences were observed between male and female patients (Figure 3F) or between primary and recurrent GBM samples (Figure 3H). However, CHID1 expression was modestly higher in older patients (>42 years) compared with younger patients (≤42 years) (p = 0.00046) (Figure 3G). These analyses from the TCGA and CGGA cohorts indicate that CHID1 expression is associated with tumor grade and patient survival in GBM. Relative to other chitinase-like family members analyzed, CHID1 showed the most consistent clinical association, supporting further investigation of its potential prognostic relevance.

### 3.3 Epigenetic Regulation, Protein Interaction Networks, and Subcellular Localization of CHID1

**Figure 4:**
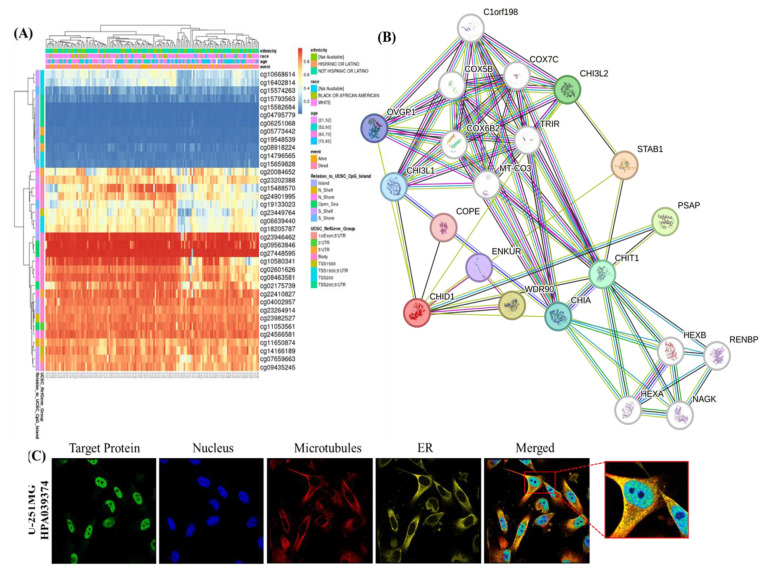
**DNA methylation, protein-protein interaction network, and subcellular localization of CHID1. (**A) Heatmap showing the DNA methylation patterns of CHID1-associated CpG sites in GBM samples, with clinical annotations displayed above the heatmap. Color gradients represent relative methylation levels across samples. (B) Protein-protein interaction (PPI) network of CHID1 and related proteins constructed using STRING, illustrating the functional associations among members of the chitinase-like family and interacting proteins. (C) Immunofluorescence images showing the subcellular localization of CHID1 protein in human cells obtained from the Human Protein Atlas database. Green indicates CHID1 staining, red represents cytoskeletal structures, and blue denotes nuclear staining (DAPI). Merged images and magnified views highlight the cellular distribution of CHID1.

To further characterize the regulatory context of CHID1 in GBM, we integrated analyses of DNA methylation, predicted protein-protein interactions (PPIs), and subcellular localization (Figure 4). DNA methylation profiling identified several CpG sites within the CHID1 locus that were inversely correlated with CHID1 mRNA expression (Pearson's r < 0, p < 0.05) (Figure 4A). Unsupervised clustering based on CHID1-associated CpG methylation patterns stratified GBM tumors into distinct groups. Tumors exhibiting lower methylation levels showed higher CHID1 expression, whereas tumors with higher methylation levels displayed reduced expression, suggesting a potential association between methylation status and transcriptional regulation. To explore the molecular interaction context of CHID1, a STRING-based PPI network was constructed (Figure 4B). Predicted interactions included several proteins associated with immune regulation and metabolic processes. Among the highest confidence interactions were STAB1 (score = 0.834), WDR90 (0.765), and PSAP (0.754). STAB1 is a scavenger receptor expressed by tumor-associated macrophages, whereas PSAP is associated with lysosomal function. Additional predicted interactions with CHI3L2, CHIA, and CHIT1 reflect intra-family relationships among chitinase-related proteins. Subcellular localization was examined using immunofluorescence data from the Human Protein Atlas in U-251 MG cells (Figure 4C). CHID1 exhibited a granular cytoplasmic distribution with enrichment in perinuclear regions. Limited overlap was observed with microtubule and endoplasmic reticulum markers, indicating predominantly cytoplasmic localization. Collectively, these analyses suggest that CHID1 expression in GBM may be influenced by DNA methylation patterns and is associated with predicted interaction networks involving immune- and metabolism-related proteins.

### 3.4 CHID1-Associated Pathways Link DNA Repair, Mitochondrial Bioenergetics, and Immune-Related Processes

**Figure 5:**
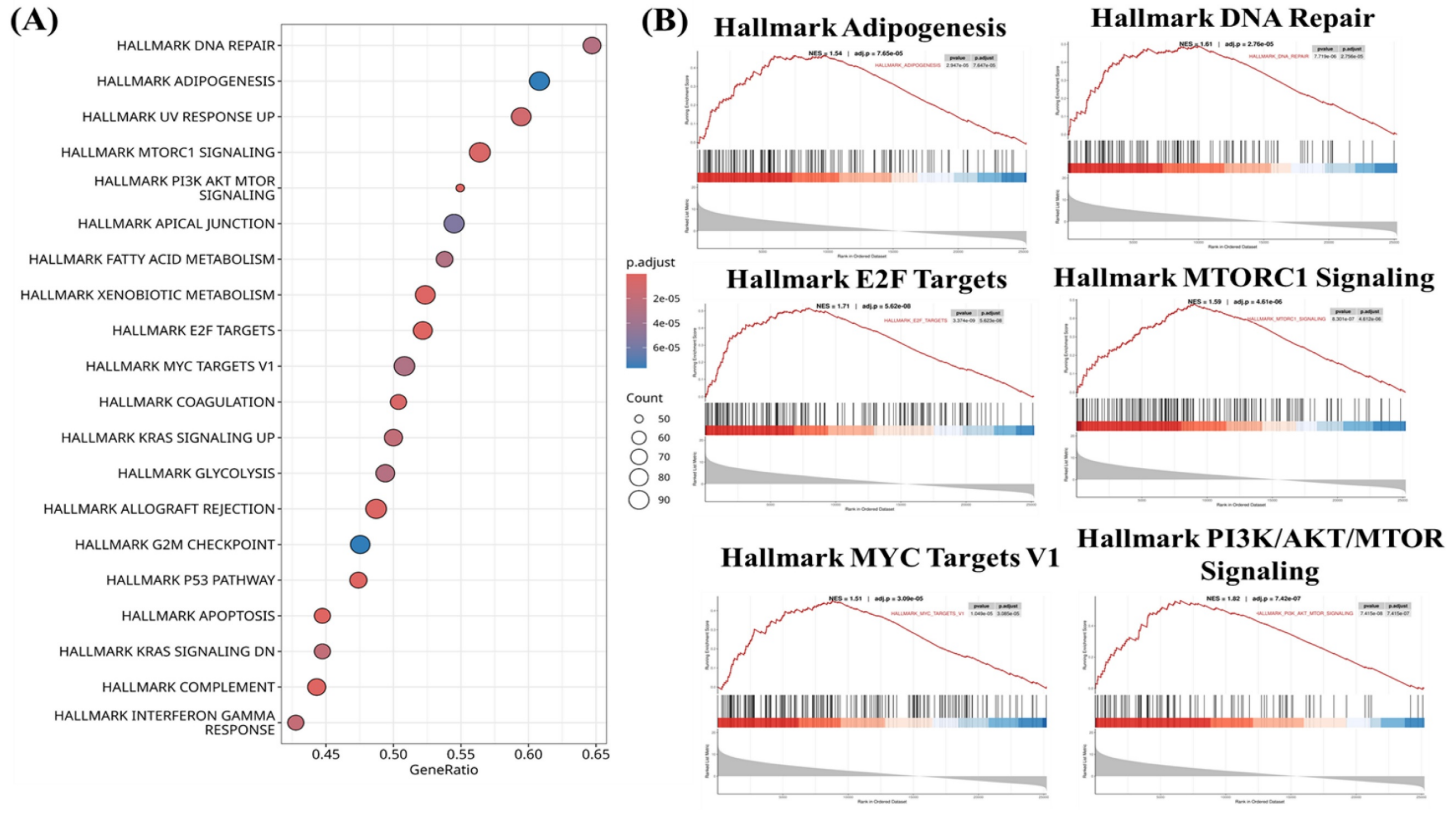
** Functional pathway enrichment associated with CHID1in GBM**. (A) Bubble plot showing Hallmark pathway enrichment analysis of genes associated with CHID1 expression in GBM. The x-axis represents the gene ratio, bubble size indicates the number of genes enriched in each pathway, and color represents the adjusted p-value. (B) Gene Set Enrichment Analysis (GSEA) plots illustrating significantly enriched Hallmark pathways associated with CHID1 expression, including adipogenesis, DNA repair, E2F targets, mTORC1 signaling, MYC targets, and PI3K-AKT-mTOR signaling. The enrichment score curves demonstrate the distribution of pathway-related genes across the ranked gene list.

**Figure 6:**
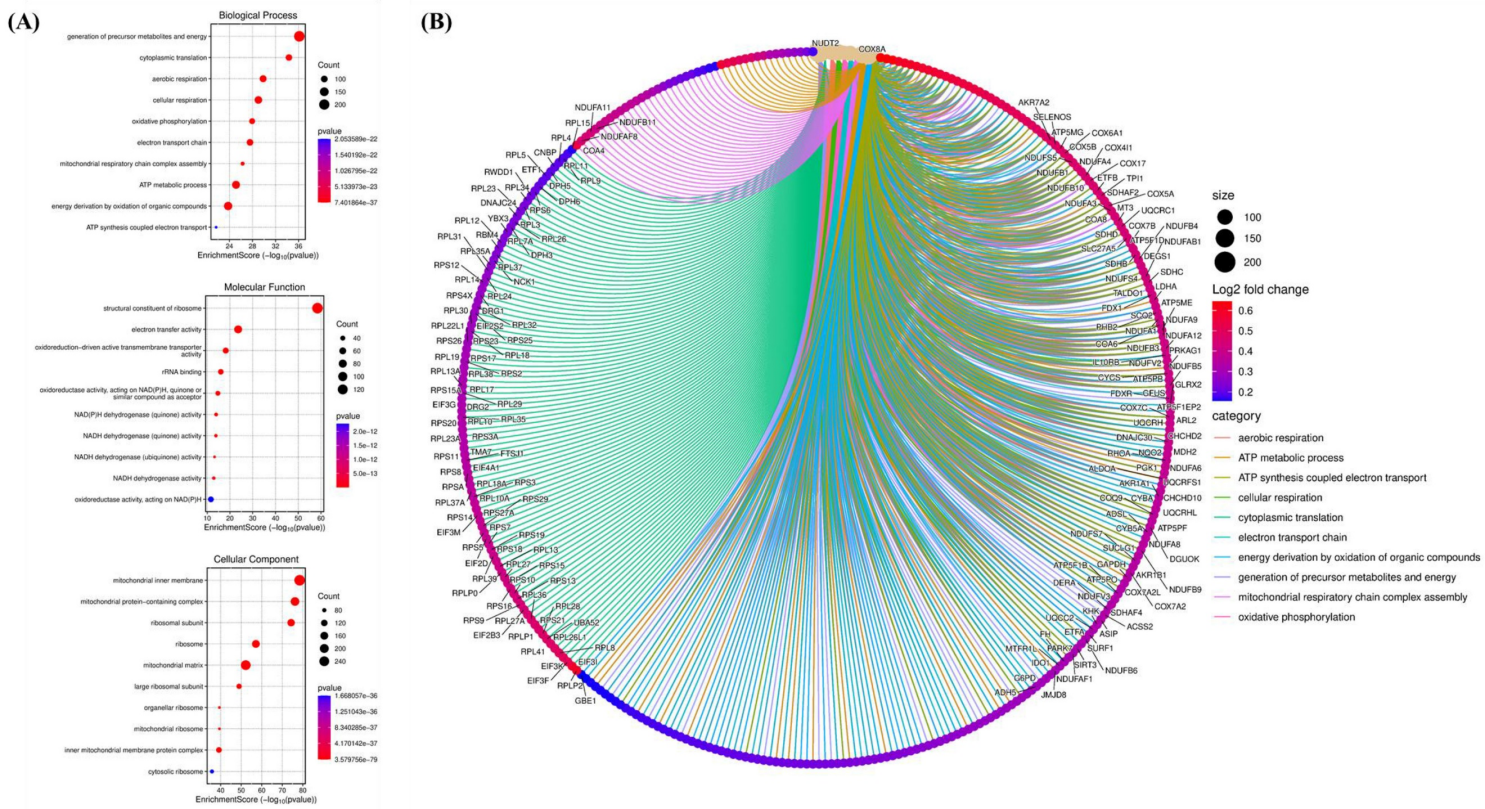
** Gene Ontology enrichment and functional interaction network associated with CHID1-related genes in GBM.** (A) Gene Ontology (GO) enrichment analysis of CHID1-associated genes, including Biological Process (BP), Molecular Function (MF), and Cellular Component (CC) categories. The bubble plots display significantly enriched terms such as oxidative phosphorylation, cellular respiration, ATP metabolic process, ribosomal structure, and mitochondrial components. Bubble size represents gene counts and color indicates statistical significance. (B) Chord diagram illustrating the relationships between enriched GO biological processes and their corresponding genes. The network highlights strong associations with mitochondrial respiration, ATP synthesis-coupled electron transport, cytoplasmic translation, and oxidative phosphorylation, indicating that CHID1-related genes are closely linked to cellular energy metabolism and mitochondrial function.

**Figure 7:**
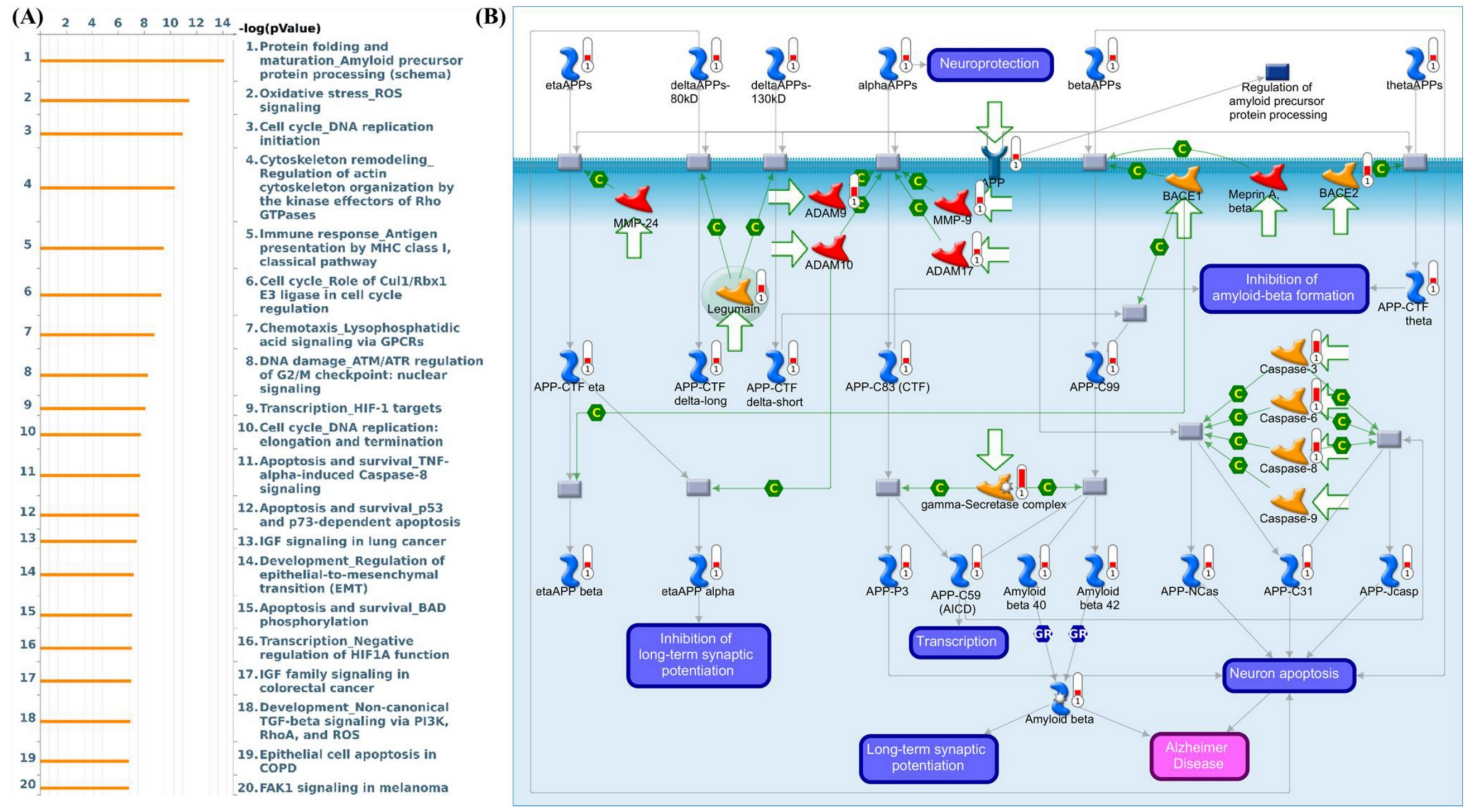
** MetaCore pathway enrichment analysis of genes associated with CHID1 expression in GBM.** (A) Bar plot showing top 20 significantly enriched biological pathways associated with CHID1-related genes identified in GBM. Pathways are ranked according to -log(p-value), highlighting processes involved in protein folding and amyloid precursor protein processing, oxidative stress response, DNA replication and cell-cycle regulation, cytoskeleton remodeling, immune response, chemotaxis signaling, DNA damage response, and apoptosis-related pathways. (B) Representative MetaCore pathway map illustrating the regulation of amyloid precursor protein (APP) processing and amyloid-beta metabolism. The network depicts interactions among key enzymes and regulatory proteins involved in APP cleavage, including ADAM family proteases, BACE1/BACE2, and the γ-secretase complex, which generate different APP cleavage fragments. The pathway also highlights downstream signaling events related to amyloid-beta production, neuronal apoptosis, transcriptional regulation, and synaptic plasticity, suggesting that CHID1-associated gene networks may participate in pathways linked to neurodegeneration-related processes, oxidative stress signaling, and cell survival mechanisms.

To characterize transcriptional programs associated with CHID1 expression in GBM, enrichment analyses were performed using gene set enrichment analysis (GSEA), Gene Ontology (GO) (Figures 5), Kyoto Encyclopedia of Genes and Genomes (KEGG) (Figures 6) and MetaCore (Figures 7) approaches. GSEA revealed significant enrichment of pathways related to DNA repair (NES = 1.61, adj. p = 2.78 × 10⁻⁵), E2F targets (NES = 1.71, adj. p = 5.62 × 10⁻⁶), and MYC targets (NES = 1.51, adj. p = 3.20 × 10⁻⁶), suggesting associations with proliferative and genome maintenance programs. Metabolic pathways including adipogenesis/fatty-acid metabolism (NES = 1.54, adj. p = 7.16 × 10⁻⁵), mTORC1 signaling (NES = 1.50, adj. p = 4.61 × 10⁻⁶), and PI3K-AKT-mTOR signaling (NES = 1.62, adj. p = 7.42 × 10⁻⁷) were also enriched. GO and KEGG analyses further indicated enrichment of mitochondrial and bioenergetic processes, including oxidative phosphorylation, ATP synthesis-coupled electron transport, and aerobic respiration. CHID1-associated genes included several mitochondrial respiratory chain components such as COX6A1, COX4I1, and SDHC. Co-expression with LDHA suggested potential links to glycolytic adaptation, whereas association with FDX1 may indicate connections to redox regulation and iron-sulfur cluster metabolism. MetaCore pathway analysis additionally highlighted pathways related to proteostasis, oxidative stress responses, antigen presentation, and DNA replication initiation. Overall, these analyses indicate that CHID1-associated transcriptional programs are enriched in pathways related to DNA repair, mitochondrial metabolism, and immune-related processes. However, these findings represent correlative pathway associations and do not establish causal functional relationships*.*

### 3.5 Single-Cell Transcriptomic Profiling Reveals CHID1 Enrichment in Glioma and Myeloid Populations

**Figure 8:**
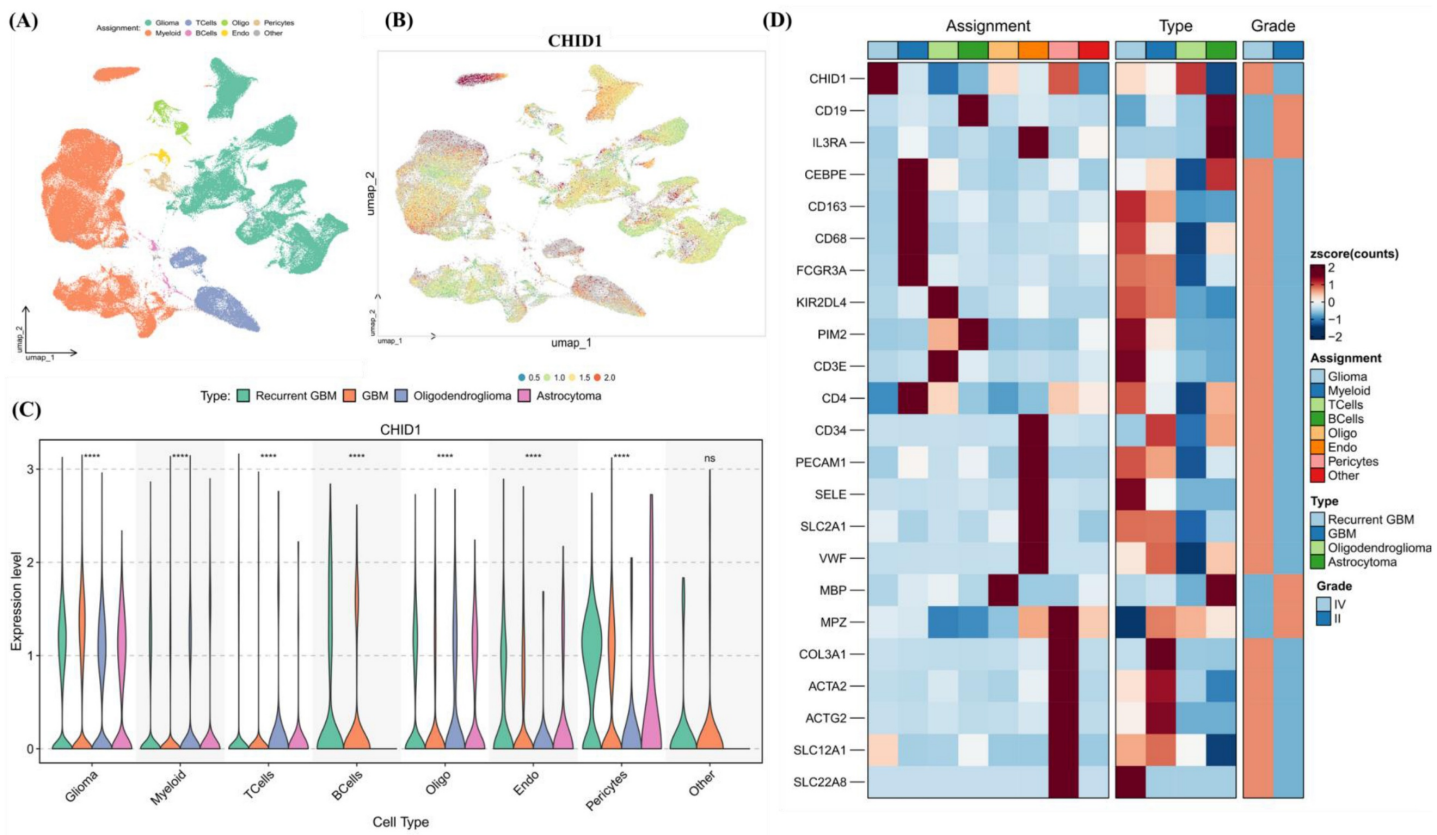
** Single-cell transcriptomic analysis of CHID1 expression across glioma cellular populations.** (A, B) UMAP visualization of single-cell RNA-seq data showing major cellular populations in glioma, including glioma cells, myeloid cells, T cells, B cells, oligodendrocytes, endothelial cells, pericytes, and other minor populations. The right panel shows the distribution of CHID1 expression projected onto the UMAP embedding. (C) Violin plots showing CHID1 expression levels across different cell types and glioma subtypes. Statistical comparisons between groups are indicated (**** p < 0.0001; ns, not significant). (D) Heatmap showing the expression of canonical marker genes across annotated cell populations, confirming the identity of major cell types. Additional annotations indicate tumor type (recurrent GBM, GBM, oligodendroglioma, and astrocytoma) and tumor grade. Expression values are shown as Z-scores.

To examine potential relationships between CHID1 expression and immune infiltration in GBM, correlation analyses were first performed using TIMER2.0. CHID1 expression showed modest negative correlations with estimated CD4⁺ T-cell infiltration (partial cor. = -0.184, p = 0.0346) and neutrophil abundance (partial cor. = -0.175, p = 0.0468) (Supplementary Figure S13). No statistically significant correlations were observed with macrophage or dendritic cell estimates. To further examine expression patterns at single-cell resolution, scRNA-seq data from the GSE182109 glioma dataset were analyzed. UMAP visualization indicated that CHID1 expression was not uniformly distributed across cell populations but appeared enriched in glioma cells and myeloid populations (Figure 8A-B). Violin plots confirmed relatively higher expression levels in these populations compared with other stromal cell types (Figure 8C). Heatmap analysis of selected immune-related genes showed that CHID1 expression clustered with several myeloid-associated markers, including CD68, CD163, and FCGR3A, as well as selected T-cell-related genes (Figure 8D). Overall, these analyses indicate that CHID1 transcripts are detectable in both malignant glioma cells and immune-related cell populations within the tumor microenvironment, although these findings describe expression distribution rather than functional interactions.

### 3.6 Integrating Pharmacogenomics and Molecular Docking to Define CHID1-Linked Drug Responses in GBM

**Figure 9:**
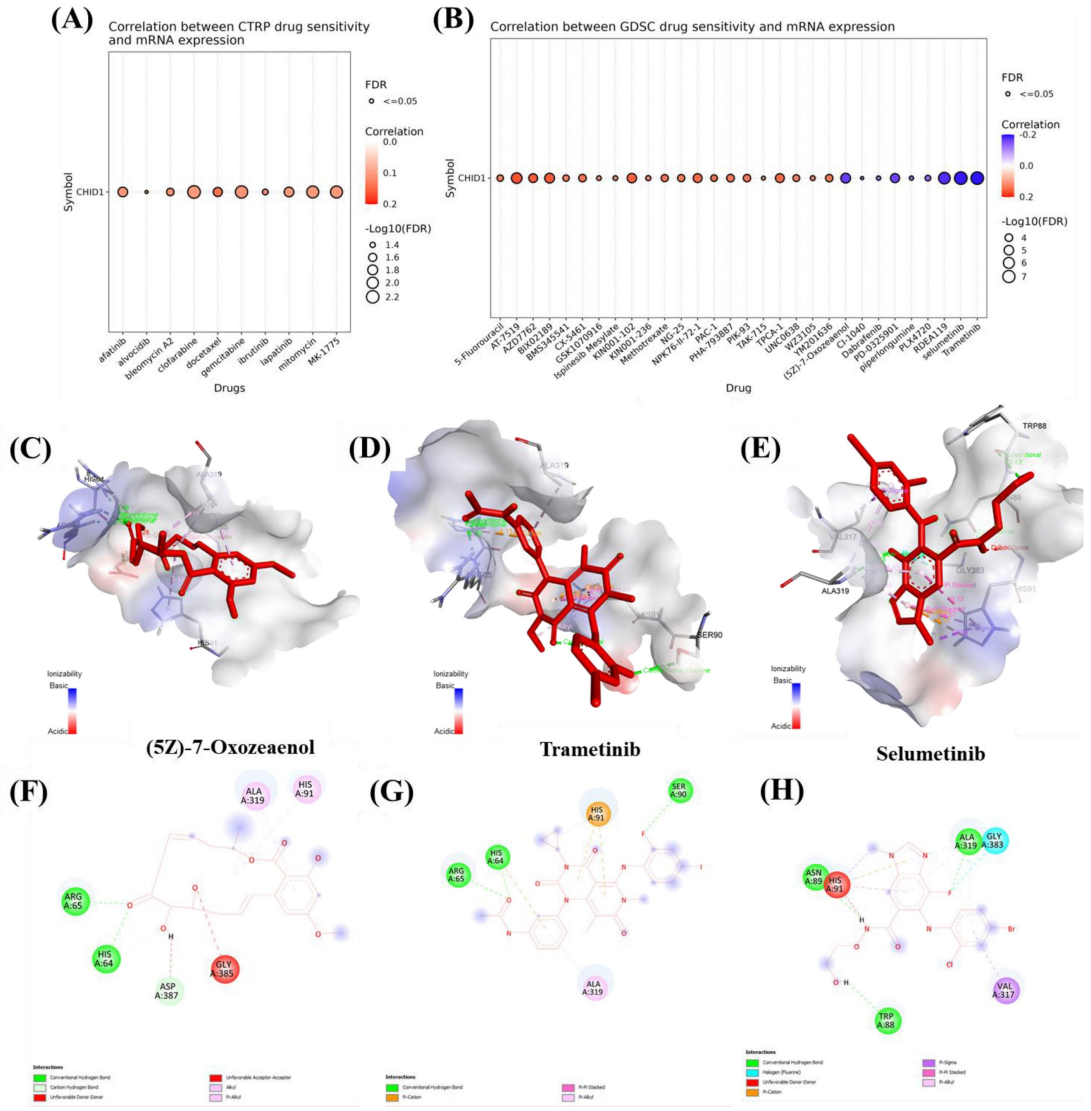
** Drug sensitivity correlation and molecular docking analysis of CHID1-associated compounds.** (A) Correlation analysis between CHID1 mRNA expression and drug sensitivity in the CTRP dataset. Bubble size represents -log10(FDR) significance, while color indicates the strength and direction of correlation. (B) Correlation between CHID1 expression and drug response in the GDSC dataset, highlighting potential therapeutic compounds associated with CHID1 expression levels. (C-E) Three-dimensional molecular docking models illustrating the predicted binding interactions between CHID1 and selected candidate compounds: (C) (5Z)-7-Oxozeaenol, (D) Trametinib, and (E) Selumetinib. The ligand molecules are shown within the predicted binding pocket of the protein. (F-H) Two-dimensional interaction diagrams showing detailed residue-ligand interactions for (F) (5Z)-7-Oxozeaenol, (G) Trametinib, and (H) Selumetinib, including hydrogen bonds, hydrophobic contacts, and other non-covalent interactions, suggesting potential binding affinity between these compounds and CHID1.

To explore potential associations between CHID1 expression and drug response patterns, pharmacogenomic datasets were analyzed alongside molecular docking simulations. Analysis of the CTRP dataset indicated modest correlations between CHID1 expression and predicted sensitivity to several agents, including gemcitabine, docetaxel, clofarabine, and the WEE1 inhibitor MK-1775 (Figure 9A). Analysis of the GDSC dataset identified both positive and negative correlations with additional compounds (Figure 9B). Positive correlations were observed with 5-fluorouracil, methotrexate, and paclitaxel, whereas negative correlations were detected for several MAPK/ERK pathway inhibitors, including trametinib, selumetinib, and (5Z)-7-oxozeaenol. To explore possible structural interactions, molecular docking simulations were performed using selected compounds. Predicted binding energies suggested potential interactions between CHID1 and (5Z)-7-oxozeaenol (-7.3 kcal/mol), trametinib (-6.8 kcal/mol), and selumetinib (-5.9 kcal/mol) (Figure 9C-E). Two-dimensional interaction diagrams illustrated predicted non-covalent contacts between these compounds and residues within the CHID1 structure (Figure 9F-H). These analyses represent computational predictions and statistical associations, and experimental validation would be required to determine whether such interactions have functional or pharmacological relevance.

### 3.7 Immunohistochemical (IHC) Validation of CHID1 Expression in Brain Tissues

To evaluate CHID1 protein expression in human tissues, immunohistochemical staining was performed on tissue microarray sections containing normal brain and brain tumor samples (Supplementary figure S14A-B). Representative images showed minimal to weak CHID1 staining in normal brain tissues, characterized by low staining intensity and a limited number of positive cells. In contrast, brain tumor specimens displayed more widespread CHID1 immunoreactivity, with a greater proportion of positively stained cells across tumor regions. Although staining intensity varied among individual cores, tumor samples generally exhibited higher CHID1 staining compared with normal brain tissues. These observations provide qualitative protein-level support for differential CHID1 expressions, but do not represent a quantitative pathological assessment.

## 4. Discussion

**Figure 10:**
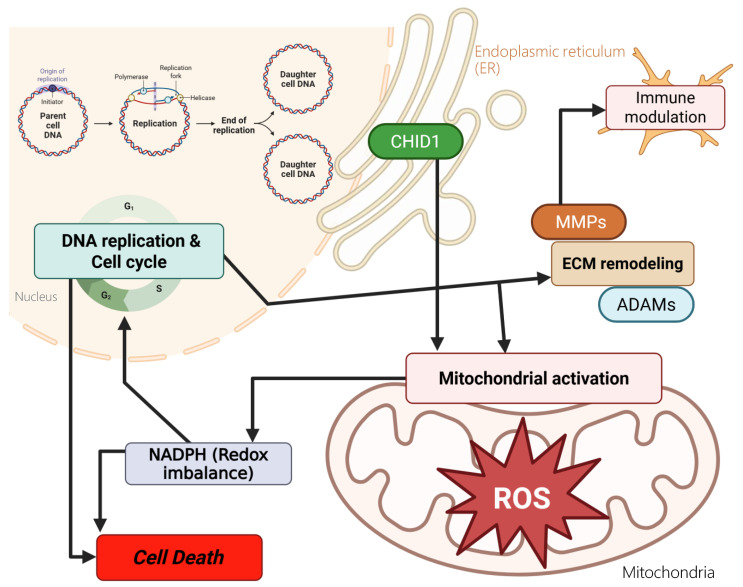
** Proposed conceptual model summarizing potential biological associations of *CHID1* in GBM.** This schematic summarizes hypothesized relationships between *CHID1* expression and metabolic and immune-related processes based on integrative transcriptomic, pathway enrichment, single-cell, and protein-level analyses. CHID1 expression was associated with pathways related to mitochondrial metabolism, oxidative phosphorylation, redox regulation, and cell cycle-related programs. Associations with immune-related pathways and cell populations are also illustrated, including patterns observed in myeloid and stromal compartments. These relationships represent putative links derived from correlative analyses and do not imply direct mechanistic regulation. The model is intended to provide a framework for future functional investigations.

GBM remains one of the most aggressive primary brain tumors and is characterized by extensive metabolic heterogeneity and a complex tumor microenvironment (Figure 10). Despite advances in molecular profiling, patient outcomes remain poor, underscoring the need to identify additional molecular features that may improve our understanding of GBM biology 77, 78. Among chitinase-like proteins, CHI3L1 has been widely studied and is recognized as a mesenchymal-associated marker in GBM. In contrast, the potential roles of other family members, including CHID1, have received comparatively less attention. This gap in the literature prompted us to examine CHID1 expression patterns in GBM using an integrative multi-dataset approach. In this study, we analyzed CHID1 expression across multiple transcriptomic datasets and complemented these analyses with pathway enrichment, single-cell transcriptomic profiling, pharmacogenomic correlations, and qualitative protein-level observations from tissue microarrays. Across independent datasets, CHID1 expression was consistently higher in GBM samples compared with normal brain tissues and showed associations with clinical outcome measures. Although CHID1 expression levels were lower than those observed for CHI3L1, the reproducible expression patterns across cohorts suggest that CHID1 may represent an additional molecular feature within the chitinase-like protein family in GBM. External validation in the GSE7696 cohort further showed that CHID1 expression was detectable across primary and recurrent tumors, including treatment-exposed samples.

Transcriptomic enrichment analyses indicated that genes associated with CHID1 expression were enriched in pathways related to DNA repair, proliferative transcriptional programs, lipid metabolism, and mTOR signaling. Complementary GO and KEGG analyses identified enrichment in oxidative phosphorylation, electron transport, and ribosome-related processes, suggesting associations with mitochondrial and biosynthetic pathways. In addition, CHID1 expression showed co-expression with several metabolic and mitochondrial genes, including FDX1, which has been implicated in iron-sulfur cluster assembly and redox-related processes 79, 80. However, these observations are based on transcriptional associations and do not establish functional relationships.

Single-cell transcriptomic analyses further indicated that CHID1 transcripts were detectable across multiple cellular compartments within GBM tissues, including glioma cells and immune-related populations. Bulk immune infiltration analysis also suggested modest correlations between CHID1 expression and selected immune cell estimates 81. These observations indicate that CHID1 expression occurs within both tumor and microenvironmental cell populations, although the biological significance of this distribution remains unclear. Similar patterns of expression across multiple cellular compartments have been described for other chitinase-like proteins, suggesting that members of this family may operate within shared biological contexts.

Pharmacogenomic analyses revealed correlations between CHID1 expression and estimated sensitivity to several therapeutic agents in large-scale drug screening datasets. These included DNA damage-targeting agents such as gemcitabine and the WEE1 inhibitor MK-1775, as well as MAPK pathway inhibitors including trametinib and selumetinib. Complementary molecular docking simulations predicted potential structural interactions between CHID1 and selected compounds, including (5Z)-7-oxozeaenol. However, these findings represent computational predictions and statistical associations derived from public datasets and therefore should be interpreted cautiously until validated experimentally 82-84.

## 5. Conclusions

This study provides an integrative analysis of CHID1 expression in GBM using transcriptomic datasets, single-cell RNA sequencing, pathway enrichment analyses, pharmacogenomic correlations, and qualitative immunohistochemical observations. CHID1 expression was consistently higher in GBM tissues compared with normal brain samples across multiple datasets and showed associations with selected clinical features. Pathway enrichment analyses suggested transcriptional programs related to mitochondrial metabolism, oxidative phosphorylation, and DNA repair. Single-cell analyses indicated that CHID1 transcripts were present in both glioma cells and immune-related populations within the tumor microenvironment. Overall, these findings describe CHID1 expression patterns in GBM and support its potential relevance as a candidate biomarker for further investigation.

## Supplementary Material

Supplementary figures and tables.

## Figures and Tables

**Table 1:** Gene annotation and genomic features of chitinase and chitinase-like family members.

Gene Symbol	Gene Name	Gene ID	Description	Chromosome Location	Synonyms
CHI3L1	Chitinase 3-like 1	1116	CHI3L1 (chitinase 3-like 1) is a protein coding gene. Diseases associated with CHI3L1 include asthma-related traits 7 and schizophrenia. Among its related pathways are innate immune system and mammary gland development pathway - Involution.	1q32.1	GP39; YKL40; YK-40
CHI3L2	Chitinase 3-like 2	1117	CHI3L2 (chitinase 3-like 2) is a protein coding gene. Diseases associated with CHI3L2 include frontotemporal dementia and/or amyotrophic lateral sclerosis 1 and frontotemporal dementia and/or amyotrophic lateral sclerosis.	1q32.1	YKL-39
CHIA	Chitinase acidic	27159	CHIA (chitinase acidic) is a protein coding gene. Diseases associated with CHIA include asthma and Gaucher's disease. Among its related pathways are digestion and absorption.	1p13.2	AMCase; TSA1902; CHIT2
CHID1	Chitinase domain containing 1	66005	CHID1 (chitinase domain containing 1) is a protein coding gene. Among its related pathways is response to elevated platelet cytosolic Ca2+.	11p15.5	MGC3234; FLJ42707; SI-CLP
CHIT1	Chitinase 1	1118	CHIT1 (chitinase 1) is a protein coding gene. Diseases associated with CHIT1 include chitotriosidase deficiency and acid phosphatase deficiency. Among its related pathways are digestion and absorption and innate immune system.	1q32.1	CHIT; CHI3

## Abbreviations

APP: amyloid precursor protein
BBB: blood-brain barrier
BP: biological process
CGGA: Chinese Glioma Genome Atlas
CHID1: chitinase domain-containing protein 1
CHI3L1: chitinase 3-like 1 (YKL-40)
CHI3L2: chitinase 3-like 2 (YKL-39)
CHIT1: chitotriosidase 1
CLP: chitinase-like protein
CTRP: Cancer Therapeutics Response Portal
DMEM: Dulbecco's modified Eagle medium
ER: endoplasmic reticulum
E2F: E2 promoter-binding factor
FBS: fetal bovine serum
FDX1: ferredoxin 1
FDR: false discovery rate
GBM: glioblastoma multiforme
GEO: Gene Expression Omnibus
GO: gene ontology
GSCA: Gene Set Cancer Analysis
GSEA: gene set enrichment analysis
IHC: immunohistochemistry
KEGG: Kyoto Encyclopedia of Genes and Genomes
MAPK: mitogen-activated protein kinase
MEK: mitogen-activated protein kinase kinase
MF: molecular function
mRNA: messenger RNA
OXPHOS: oxidative phosphorylation
PI3K: phosphatidylinositol 3-kinase
PPI: protein-protein interaction
RNA-Seq: RNA sequencing
ROS: reactive oxygen species
scRNA-Seq: single-cell RNA sequencing
TAM: tumor-associated macrophage
TCA: tricarboxylic acid
TCGA: The Cancer Genome Atlas
TME: tumor microenvironment
UMAP: Uniform Manifold Approximation and Projection
