# Supplementary material for: Integrative Multi-Omics and Single-Cell Profiling Identify Chitinase Domain Containing Protein 1 (CHID1) as a Prognostic Biomarker in Glioblastoma
Source: J Cancer. 2026 Mar 17;17(3):662–78. doi: 10.7150/jca.130519 (PMC13003607; doi:10.7150/jca.130519)
Supplement: Supplementary file 1 — Supplementary figures and tables. [file jcav17p0662s1.pdf]

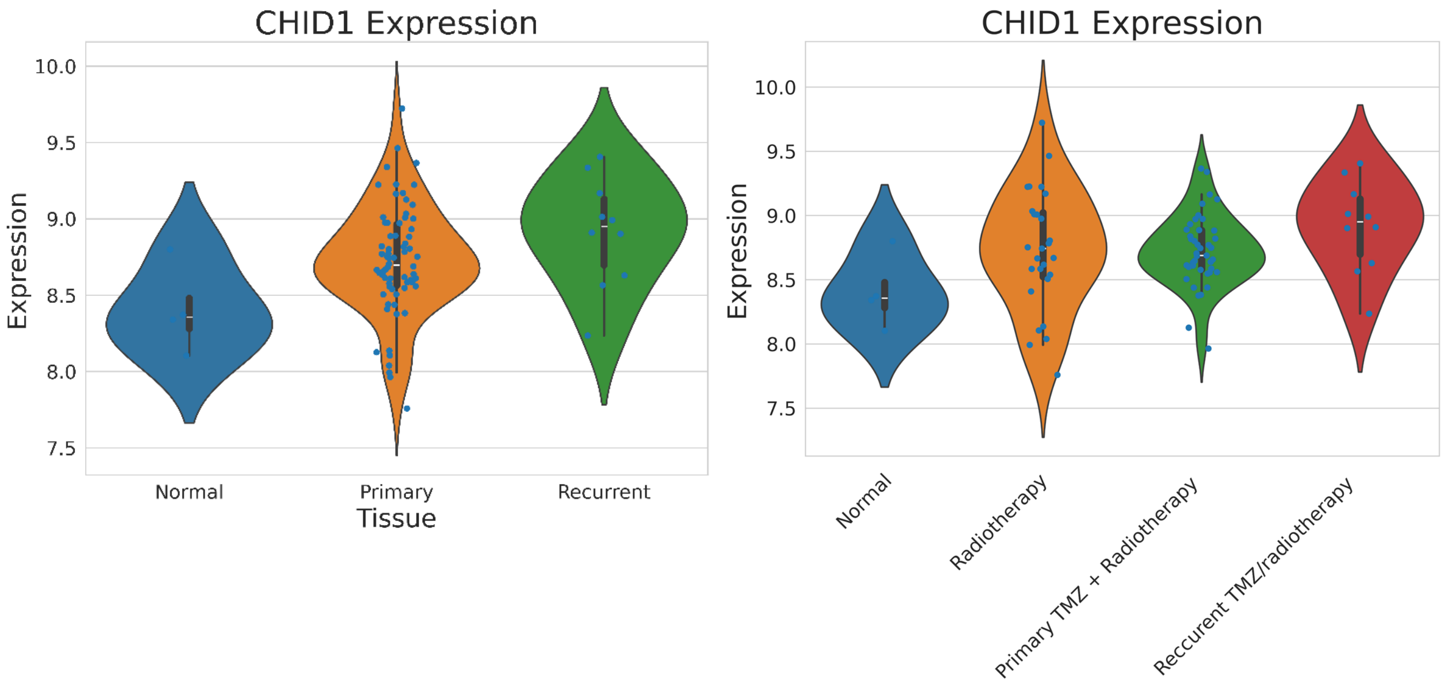

Supplementary Figure S1. CHID1 expression in GBM tissues and treatment subgroups from GSE7696. (A) Violin plots showing CHID1 expression across normal brain, primary GBM, and recurrent GBM tissues. Expression of CHID1 is elevated in primary GBM compared to normal tissue and further increases in recurrent GBM. (B) Violin plots stratified by treatment subgroups. CHID1 expression remains significantly higher in GBM samples receiving radiotherapy alone, TMZ + radiotherapy (primary), and recurrent TMZ/radiotherapy-treated tumors compared with normal brain tissues. These findings suggest that CHID1 expression is sustained or enhanced following standard-of-care treatments, potentially contributing to therapy resistance and recurrence in GBM.

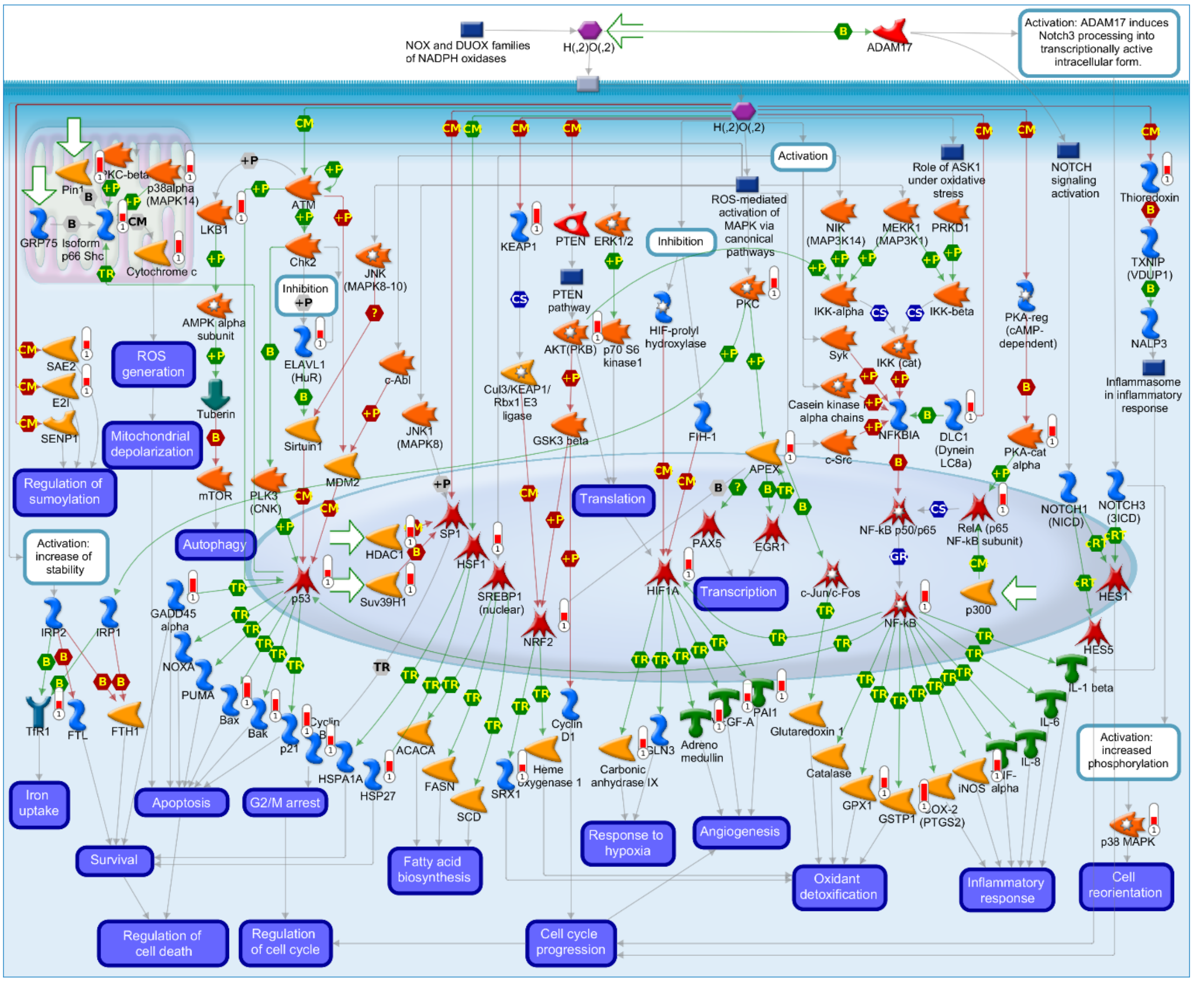

Supplementary Figure S2. MetaCore enrichment pathway (Oxidative stress_ROS signaling) analysis of genes co-expressed with CHID1

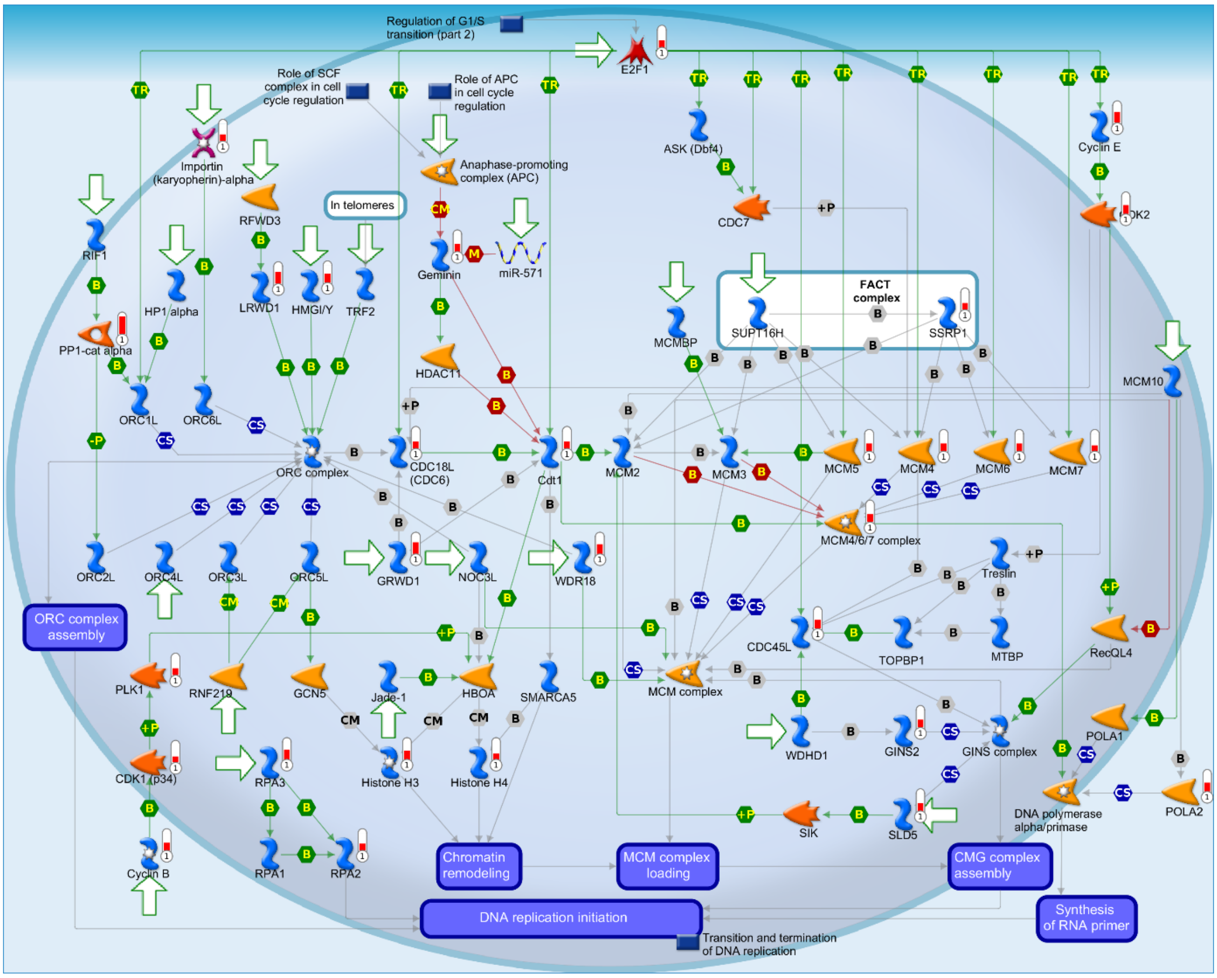

Supplementary Figure S3. MetaCore enrichment pathway (Cell cycle_DNA replication initiation) analysis of genes co-expressed with CHID1

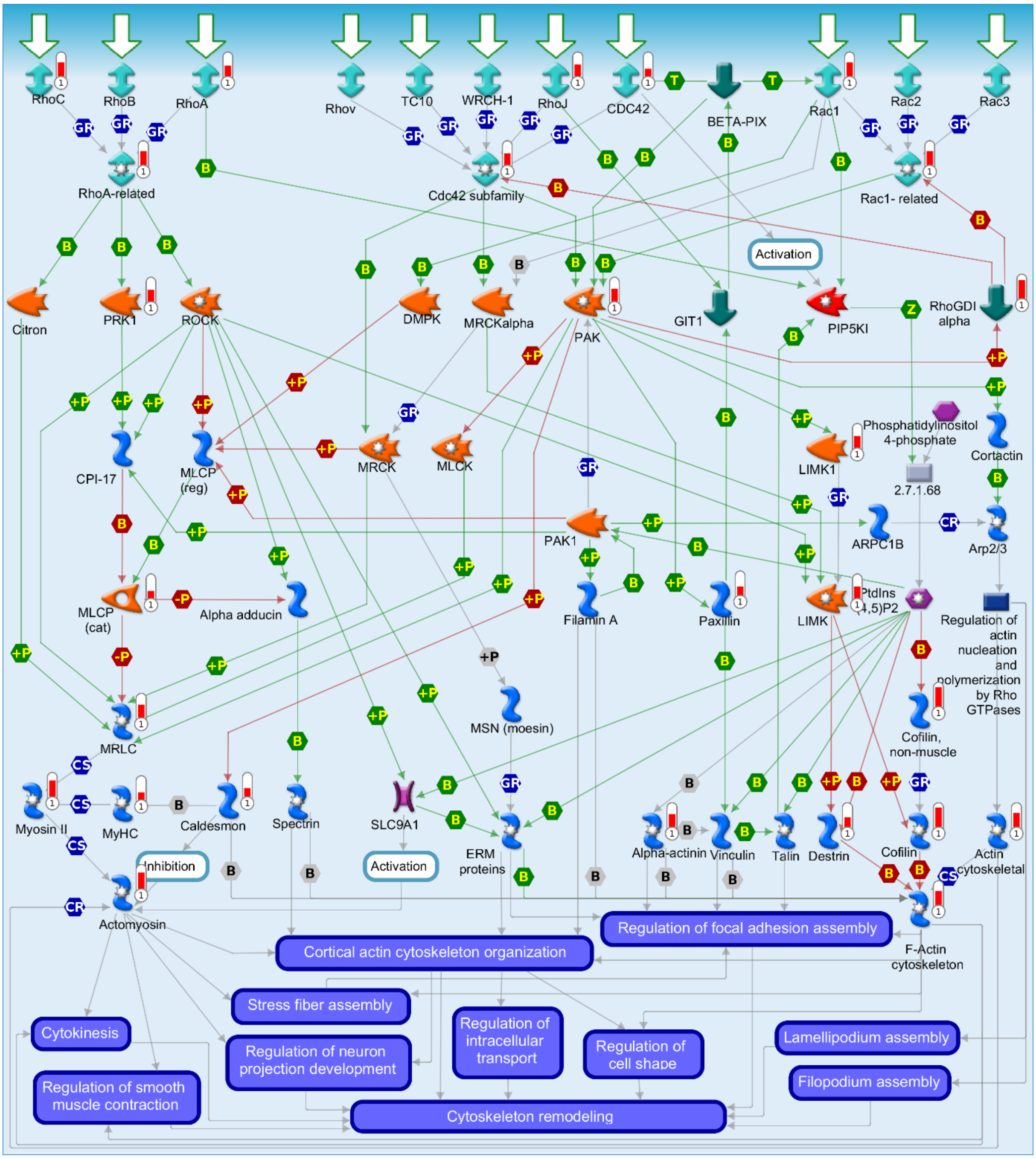

Supplementary Figure S4. MetaCore enrichment pathway (Cytoskeleton remodeling_Regulation of actin cytoskeleton organization by the kinase effectors of Rho GTPases) analysis of genes co-expressed with CHID1

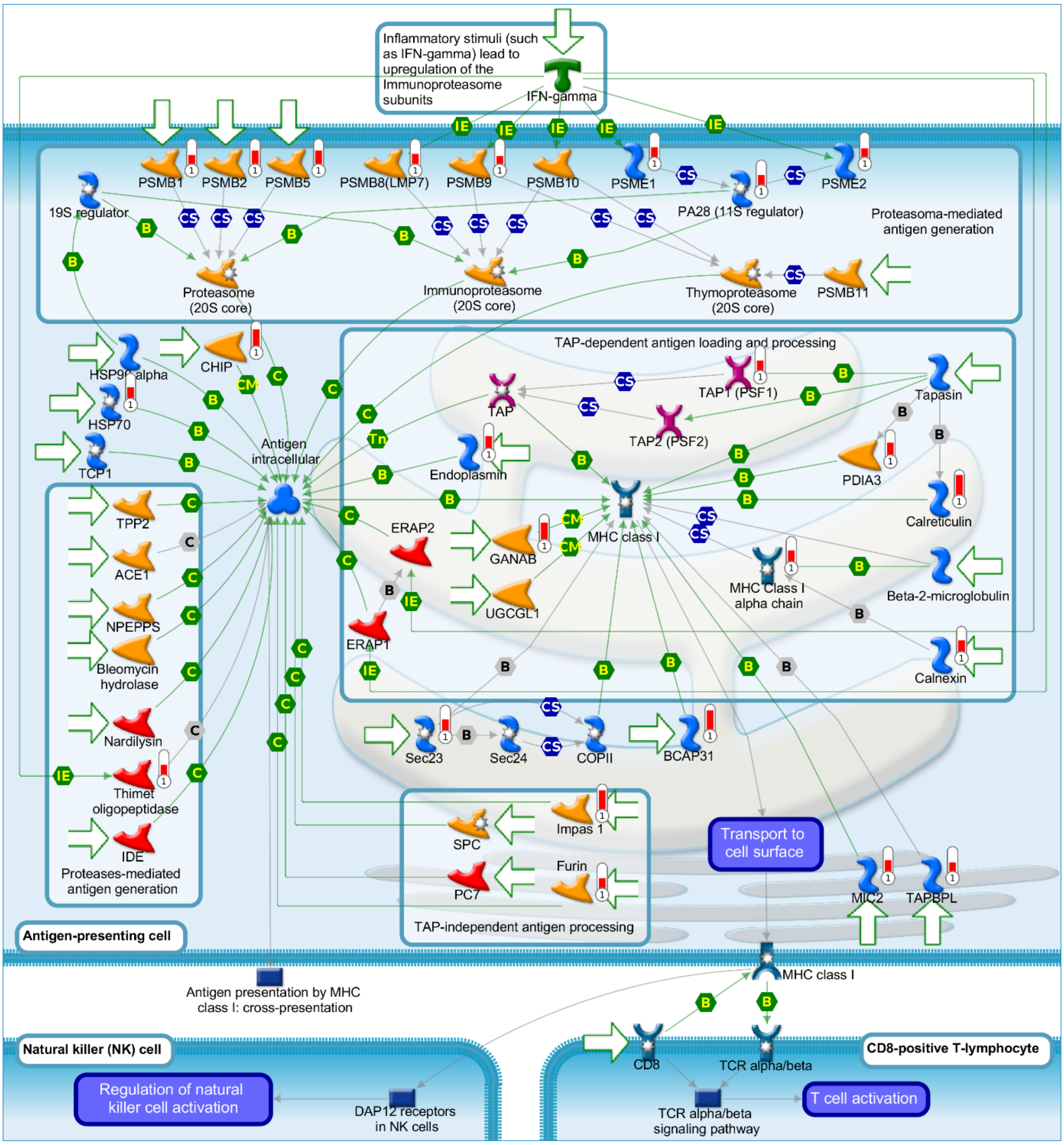

Supplementary Figure S5. MetaCore enrichment pathway (Immune response_Antigen presentation by MHC class I, classical pathway) analysis of genes co-expressed with CHID1

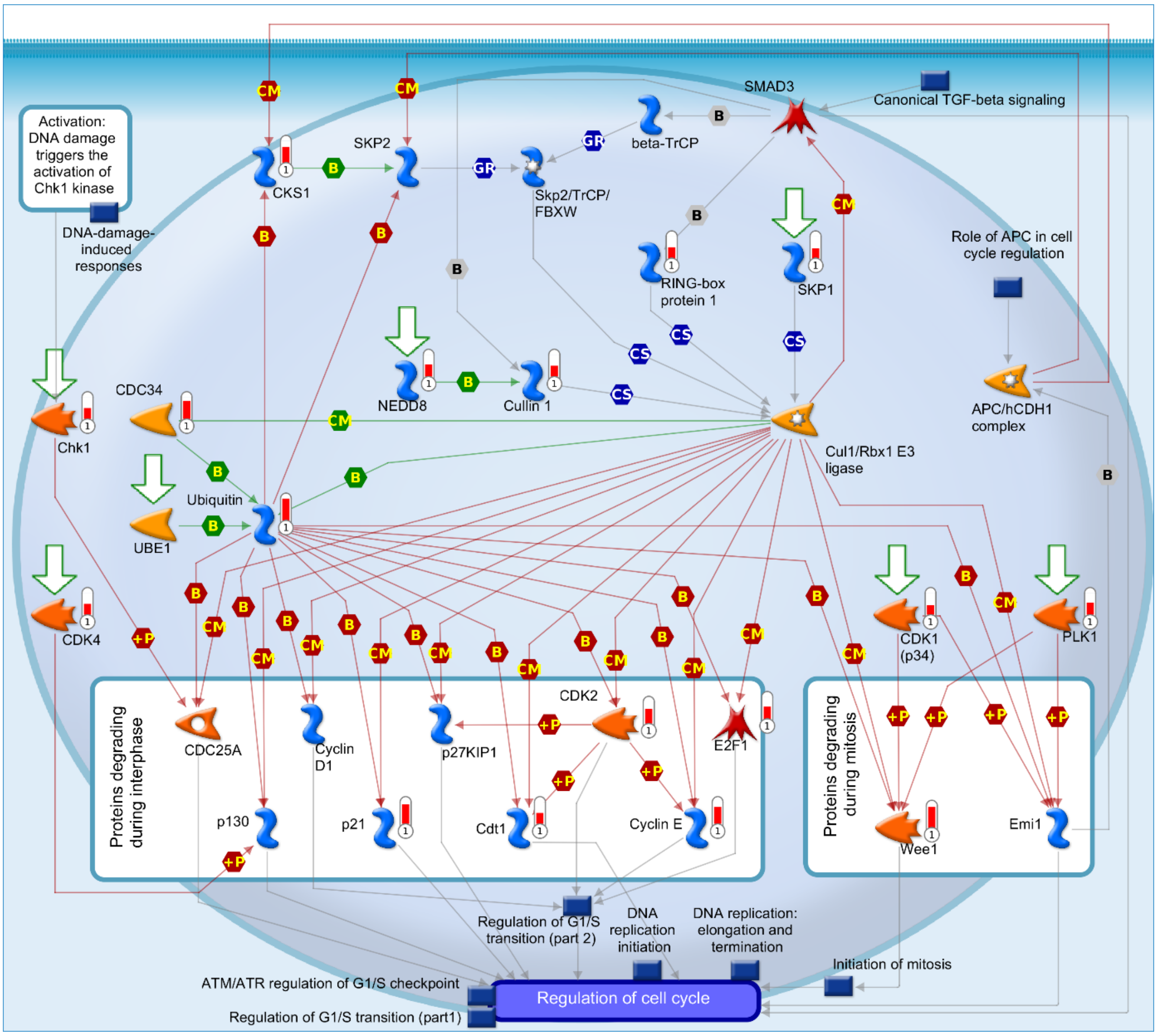

Supplementary Figure S6. MetaCore enrichment pathway (Cell cycle_Role of Cul1/Rbx1 E3 ligase in cell cycle regulation) analysis of genes co-expressed with CHID1

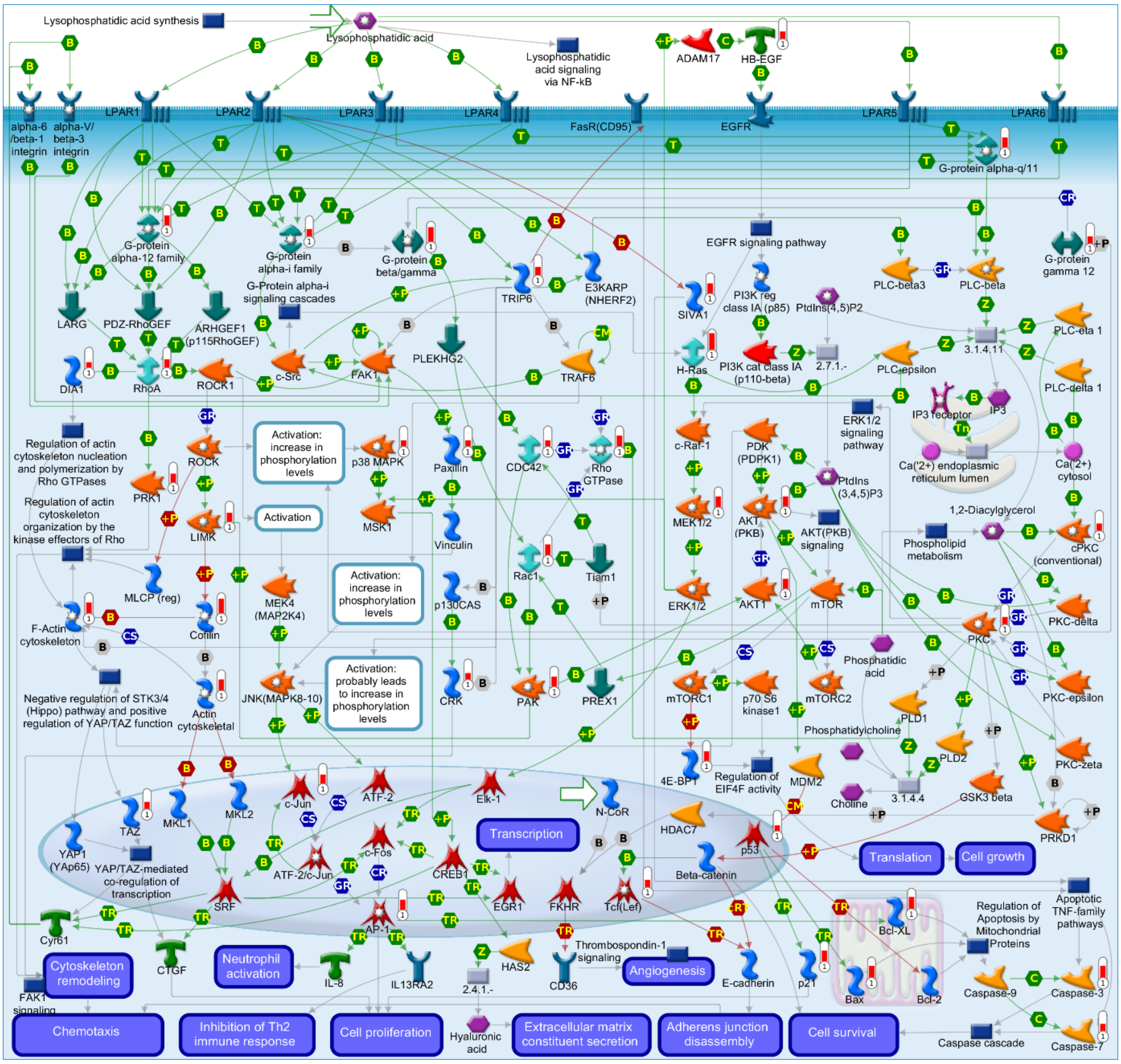

Supplementary Figure S7. MetaCore enrichment pathway (Chemotaxis_Lysophosphatidic acid signaling via GPCRs) analysis of genes co-expressed with CHID1

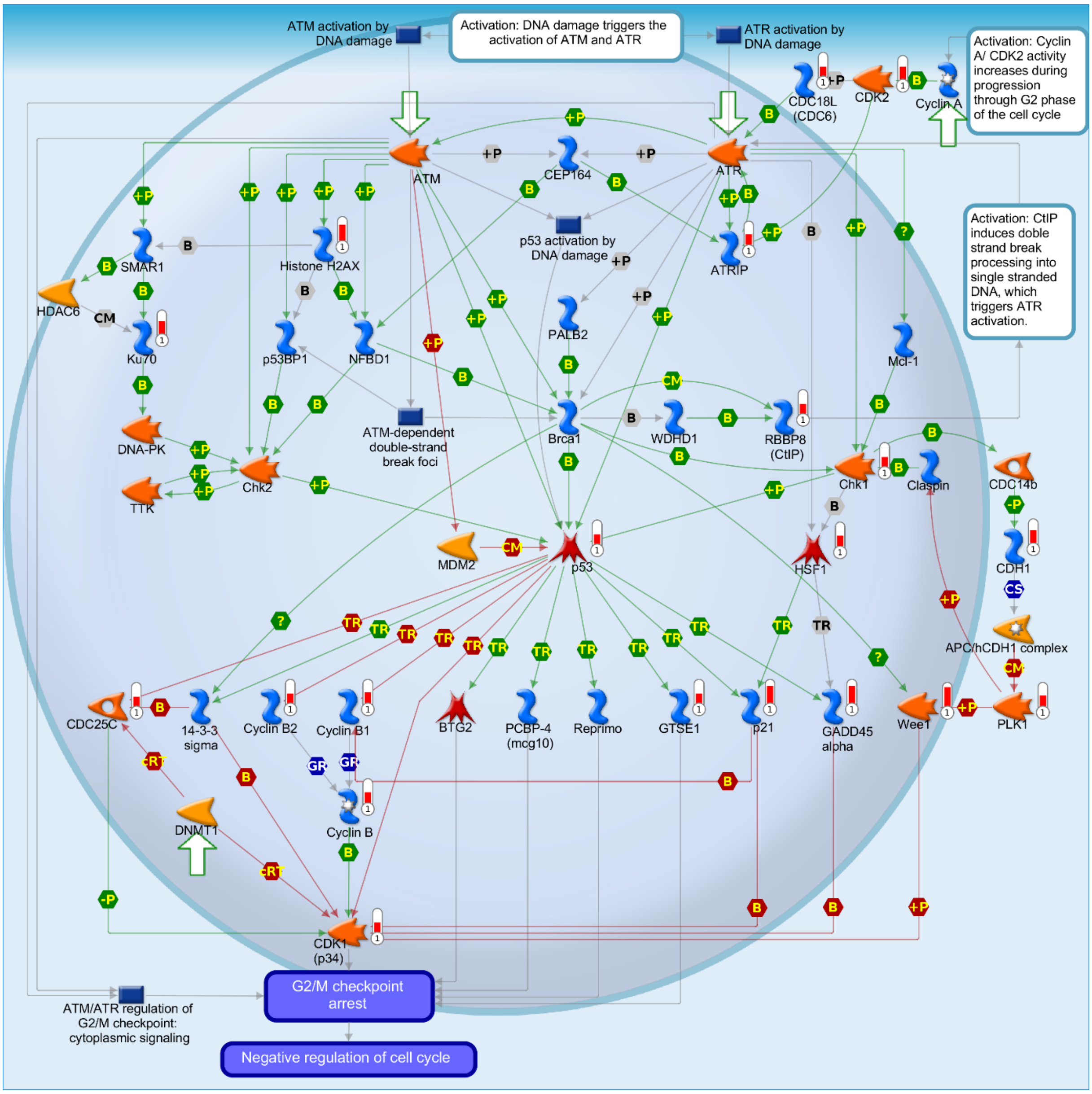

Supplementary Figure S8. MetaCore enrichment pathway (DNA damage_ATM/ATR regulation of G2/M checkpoint: nuclear signaling) analysis of genes co-expressed with CHID1

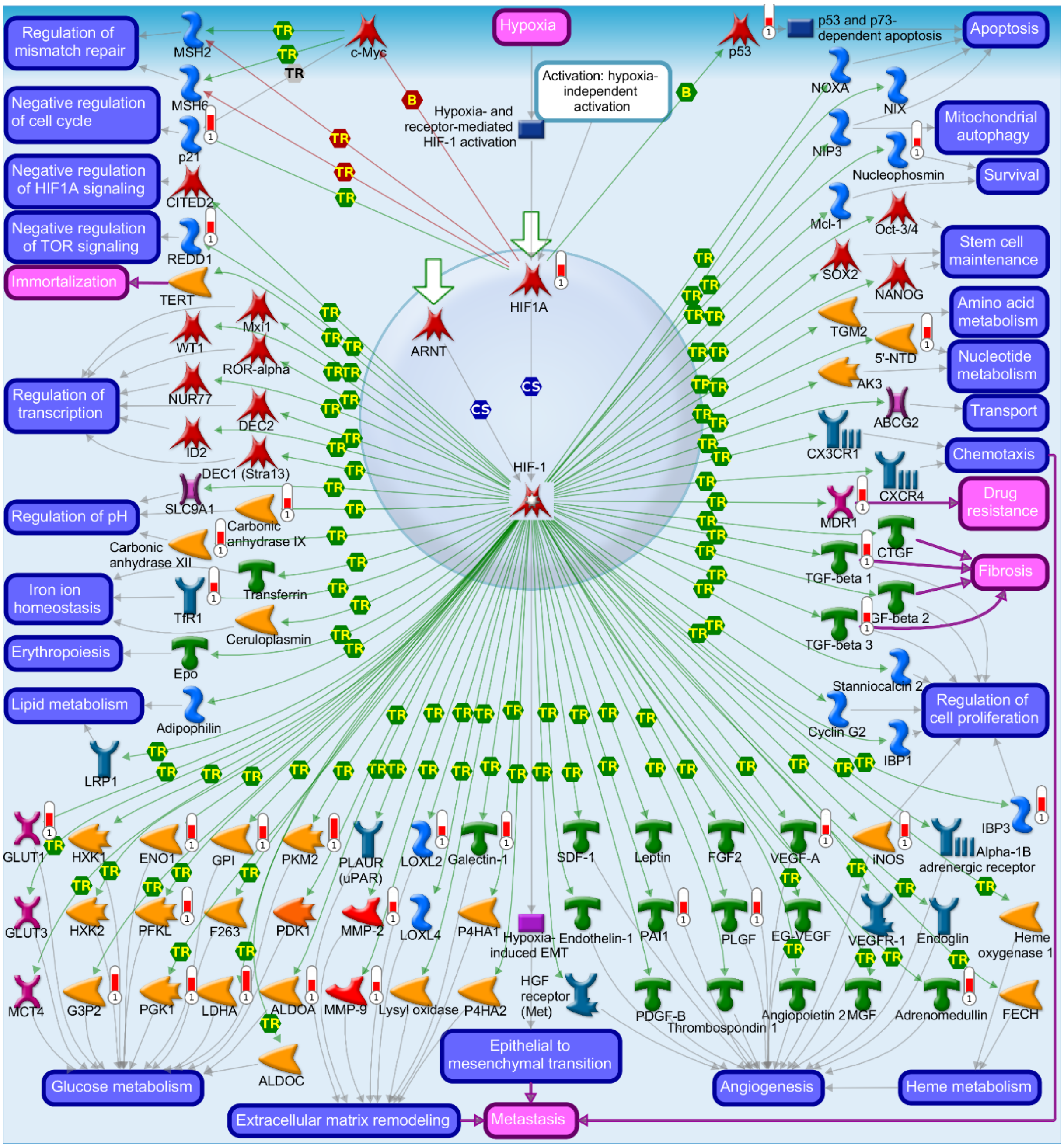

Supplementary Figure S9. MetaCore enrichment pathway (Transcription_HIF-1 targets) analysis of genes co-expressed with CHID1

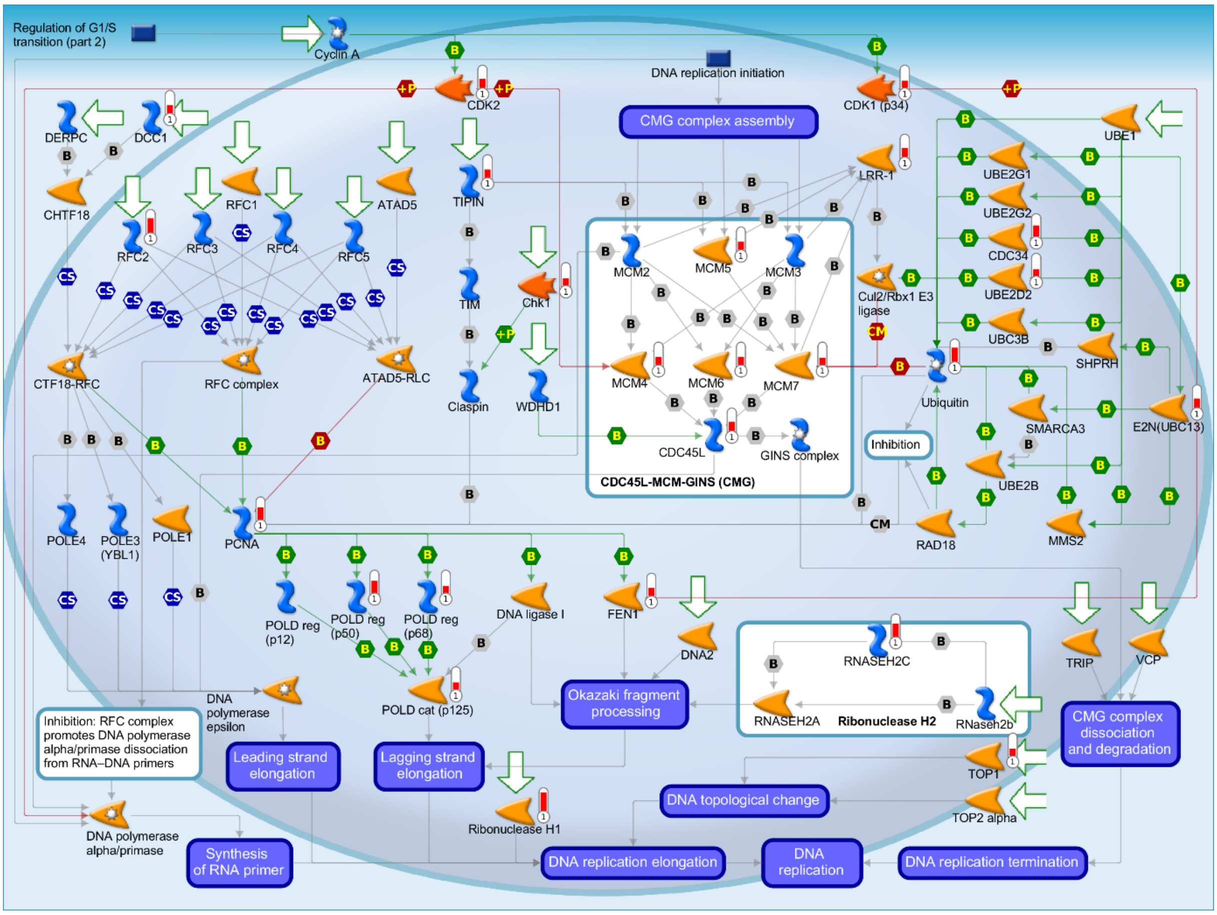

Supplementary Figure S10. MetaCore enrichment pathway (Cell cycle_DNA replication elongation and termination) analysis of genes co-expressed with CHID1

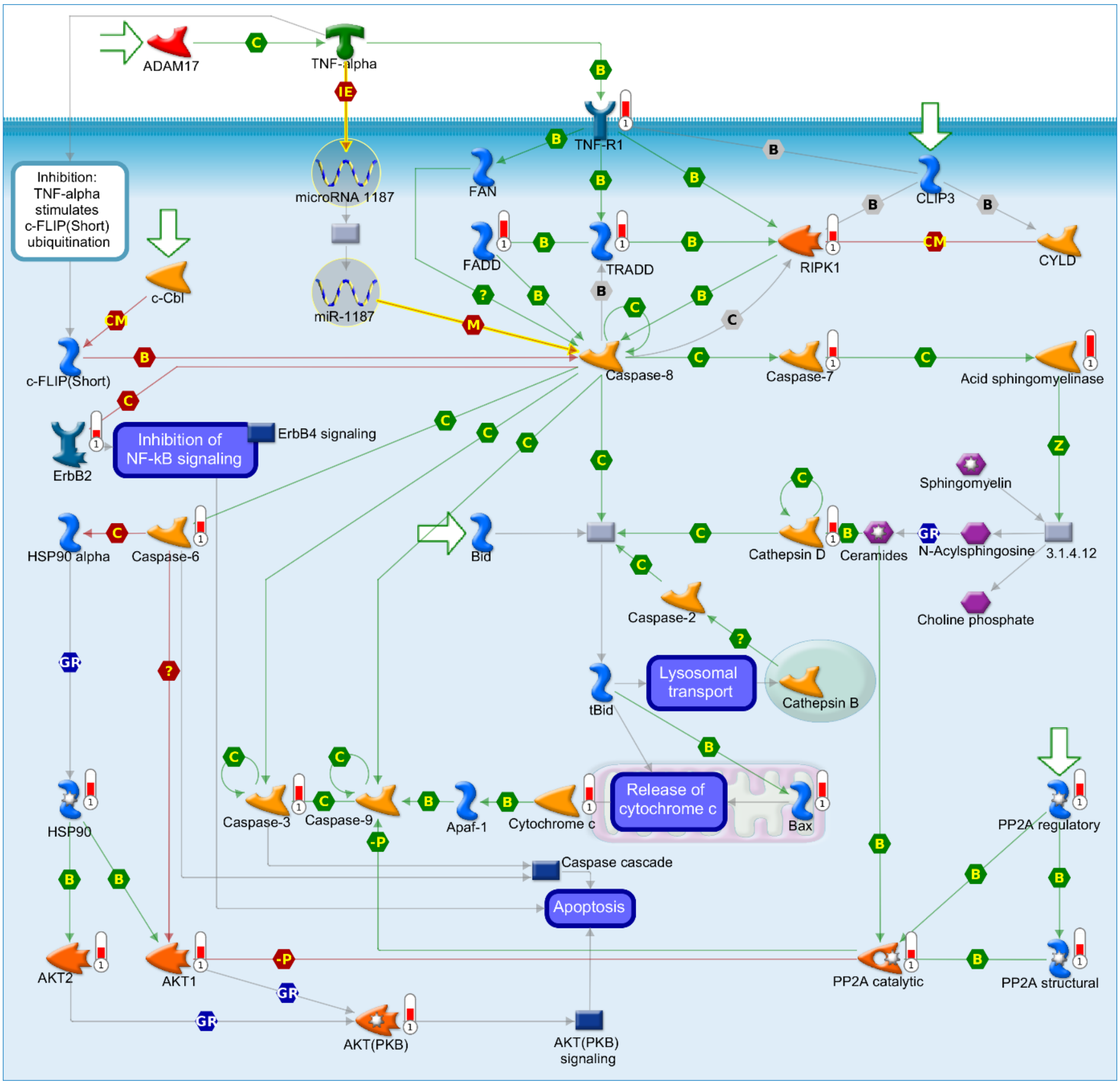

Supplementary Figure S11. MetaCore enrichment pathway (Apoptosis and survival_TNF-alpha-induced Caspase-8 signaling) analysis of genes co-expressed with CHID1

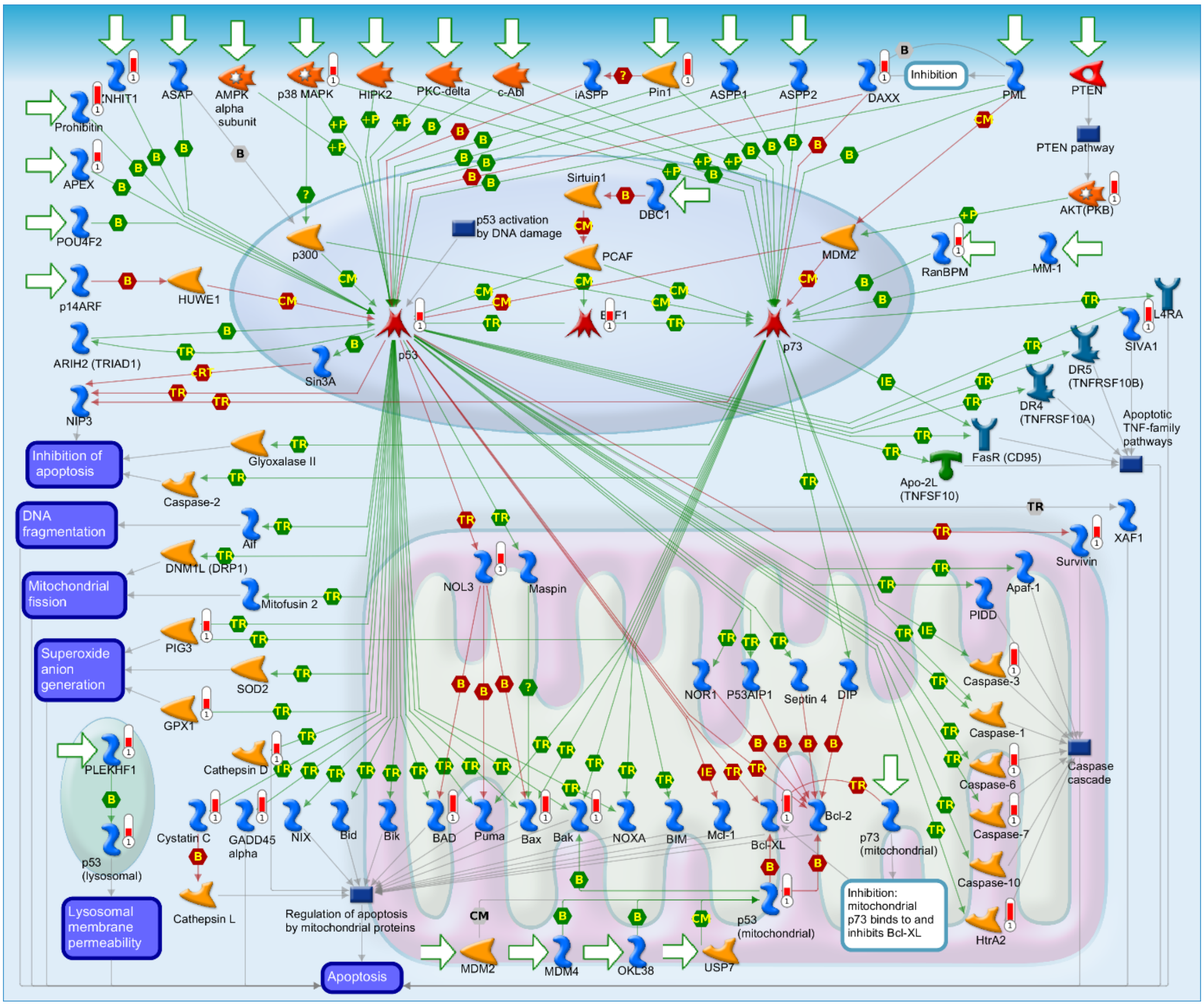

Supplementary Figure S12. MetaCore enrichment pathway (Apoptosis and survival_p53 and p73-dependent apoptosis) analysis of genes co-expressed with CHID1

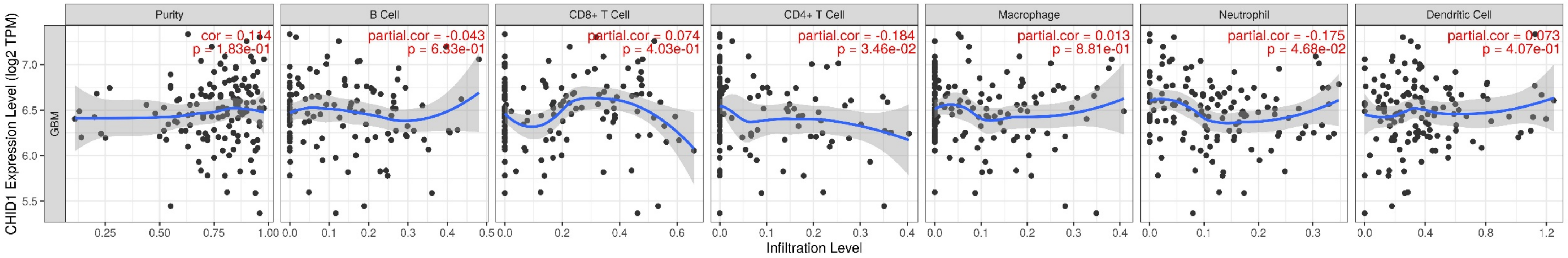

Supplementary Figure S13. Correlation between CHID1 expression and immune cell infiltration in GBM using TIMER2.0. Scatter plots show partial Spearman's correlation between CHID1 expression (log2 TPM) and infiltration levels of (A) tumor purity, (B) B cells, (C) $CD8^{+}$ T cells, (D) $CD4^{+}$ T cells, (E) macrophages, (F) neutrophils, and (G) dendritic cells. Blue regression lines represent the fitted correlation trend, and shaded areas indicate 95% confidence intervals. Correlation coefficients (cor) and p-values are indicated in red. Significant negative correlations were observed with $CD4^{+}$ T cells (partial cor = –0.184, $p = 3.46 \times 10^{-2}$) and neutrophils (partial cor = –0.175, $p = 4.68 \times 10^{-2}$), suggesting that CHID1 expression may contribute to immunosuppressive remodeling of the GBM microenvironment.

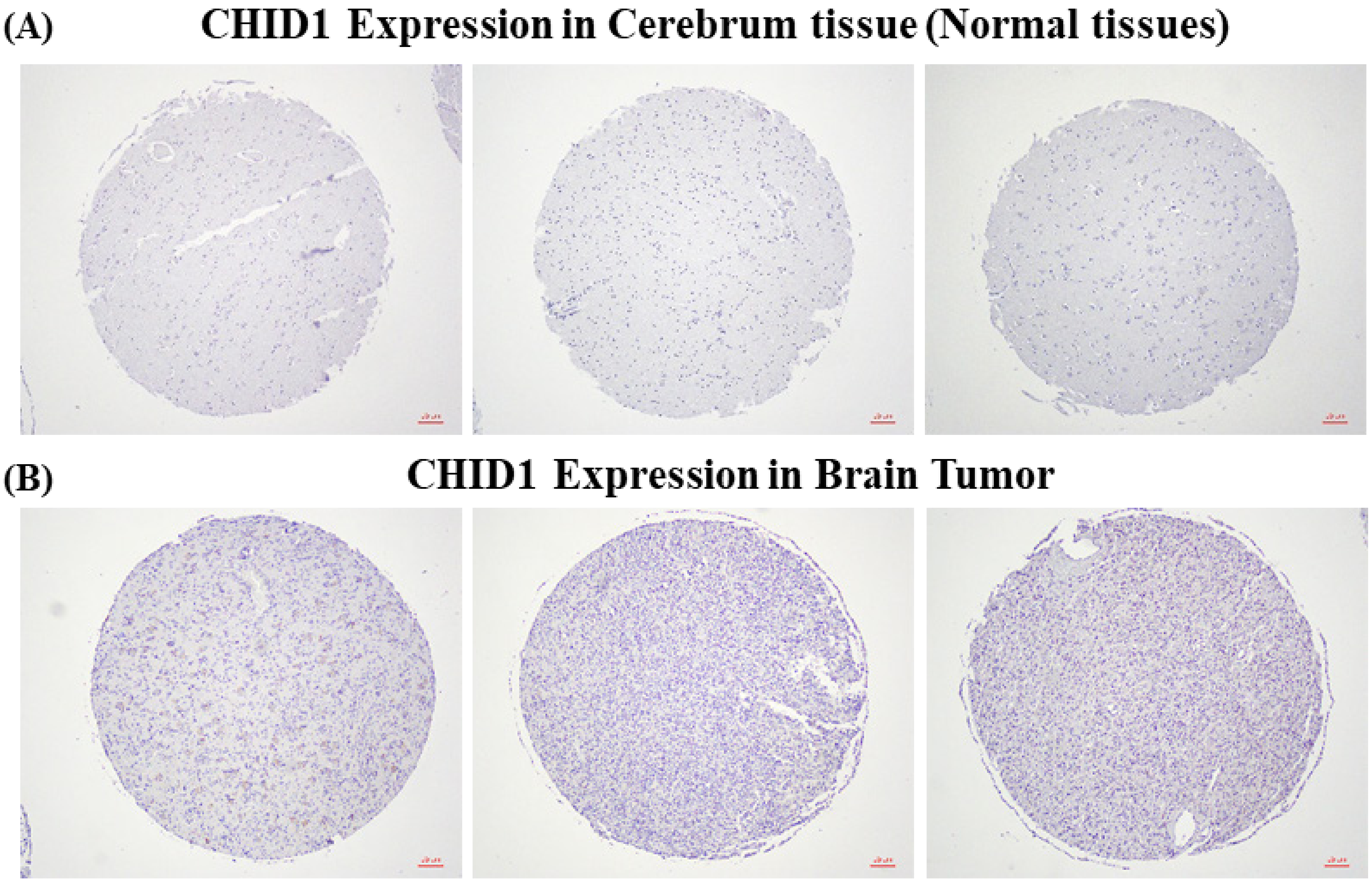

Supplementary Figure S14. Immunohistochemical staining of CHID1 in human brain tumor tissues. (A-B) Representative immunohistochemical images showing CHID1 staining in normal brain tissues and brain tumor specimens. Normal brain tissues show low CHID1 staining, whereas tumor tissues show more-apparent staining signal across tissue sections. All images were acquired at the same magnification. Scale bar, 50 μm.

Table S1. **Protein–protein interaction (PPI) partners of CHID1 identified by STRING analysis**.The table lists the top predicted CHID1 interactors (Node 2) along with their interaction confidence scores. Interactions include members of the chitinase family (CHI3L1, CHI3L2, CHIA, CHIT1), mitochondrial/energy-related proteins (COPE, ENKUR, OVGP1, WDR90), and immune/metabolic regulators (PSAP, STAB1). The interaction scores (range: 0–1) reflect the strength of association, with higher scores indicating stronger predicted confidence.

Node 1	**Node 2**	**Score**
CHID1	CHI3L1	0.599
CHID1	CHI3L2	0.745
CHID1	CHIA	0.665
CHID1	CHIT1	0.683
CHID1	COPE	0.518
CHID1	ENKUR	0.545
CHID1	OVGP1	0.564
CHID1	PSAP	0.754
CHID1	STAB1	0.84
CHID1	WDR90	0.765

Table **S2. Pathway analysis of gene co-expressed CHID1 from the MetaCore** database (with *p*-value< 0.05 set as the cutoff value).

#	Maps	pValue	Min FDR	Network Objects from Active Data
1	Protein folding and maturation_Amyloid precursor protein processing (schema)	8.046E-15	1.205E-11	APP-C31, APP-CTF delta-short, gamma-Secretase complex, APP-CTF delta-long, betaAPPs, ADAM9, deltaAPPs-80kD, Caspase-6, APP-P3, alphaAPPs, etaAPP beta, APP-CTF theta, Caspase-3, APP-C99, APP-C83 (CTF), etaAPP alpha, APP-NCas, Amyloid beta 40, APP, APP-CTF eta, thetaAPPs, Amyloid beta, etaAPPs, MMP-9, APP-C59 (AICD), deltaAPPs-130kD, APP-Jcasp, Amyloid beta 42
2	Oxidative stress_ROS signaling	4.206E-12	3.150E-09	GSTP1, TfR1, ELAVL1 (HuR), p38alpha (MAPK14), HSF1, Thioredoxin, Bax, RelA (p65 NF-kB subunit), iNOS, GPX1, PKC, LKB1, KEAP1, Adrenomedullin, PKA-cat alpha, HIF1A, SRX1, HSP27, DLC1 (Dynein LC8a), GADD45 alpha, VEGF-A, HDAC1, Pin1, NF-kB, SAE2, AKT(PKB), Isoform p66 Shc, p21, Bak, Cytochrome c, Cyclin B1, p53, NRF2, E2I, p38 MAPK, APEX, PAI1, Carbonic anhydrase IX, Suv39H1
3	Cell cycle_DNA replication initiation	1.331E-11	6.647E-09	CDC18L (CDC6), WDR18, MCM7, Cyclin B, Histone H3, GINS2, MCM5, RPA3, SLD5, MCM4, Cyclin E, E2F1, PP1-cat alpha, Importin (karyopherin)-alpha, MCM6, POLA2, HMGI/Y, SSRP1, MCM4/6/7 complex, GRWD1, Cdt1, Geminin, PLK1, RPA2, Histone H4, LRWD1, CDK1 (p34), CDK2, CDC45L
4	Cytoskeleton remodeling_Regulation of actin cytoskeleton organization by the kinase effectors of Rho GTPases	5.826E-11	2.182E-08	MLCP (cat), RhoA, Actomyosin, PAK, Cofilin, non-muscle, Rac1-related, Cdc42 subfamily, RhoJ, Destrin, F-Actin cytoskeleton, MRLC, PRK1, RhoA-related, Myosin II, Caldesmon, CDC42, Actin cytoskeletal, Alpha-actinin, RhoC, LIMK1, MyHC, LIMK, Paxillin, Cofilin, Rac1, RhoGDI alpha
5	Immune response_Antigen presentation by MHC class I, classical pathway	3.975E-10	1.191E-07	MHC Class I alpha chain, TAP1 (PSF1), PDIA3, MIC2, GANAB, PSMB8(LMP7), PSME2, Endoplasmin, Calreticulin, PSMB2, Thimet oligopeptidase, PSMB1, PSMB5, PSMB9, Calnexin, PA28 (11S regulator), TAPBPL, Sec23, CHIP, HSP70, Furin, BCAP31, Impas 1, PSME1
6	Cell cycle_Role of Cul1/Rbx1 E3 ligase in cell cycle regulation	6.148E-10	1.535E-07	Cullin 1, Ubiquitin, Chk1, Wee1, CKS1, CDK4, Cyclin E, E2F1, NEDD8, CDC34, RING-box protein 1, p21, Cdt1, SKP1, PLK1, CDK1 (p34), CDK2
7	Chemotaxis_Lysophosphatidic acid signaling via GPCRs	2.317E-09	4.958E-07	RhoA, 4E-BP1, cPKC (conventional), G-protein alpha-12 family, Bax, AP-1, Caspase-7, PAK, AKT1, PKC, DIA1, Tcf(Lef), G-protein alpha-i family, H-Ras, F-Actin cytoskeleton, Caspase-3, c-Jun, PRK1, SIVA1, HB-EGF, Bcl-XL, MEK1/2, G-protein beta/gamma, Rho GTPase, CDC42, Actin cytoskeletal, TAZ, CRK, AKT(PKB), G-protein alpha-q/11, p21, p53, LIMK, TRIP6, p38 MAPK, Paxillin, Cofilin, Rac1, G-protein gamma 12
8	DNA damage_ATM/ATR regulation of G2/M checkpoint: nuclear signaling	7.158E-09	1.340E-06	CDC18L (CDC6), HSF1, Chk1, Wee1, Cyclin B, Cyclin B2, CDC25C, GTSE1, CDH1, Histone H2AX, GADD45 alpha, Ku70, p21, RBBP8

				(CtIP), Cyclin B1, p53, PLK1, ATRIP, CDK1 (p34), CDK2
9	Transcription_HIF-1 targets	1.114E-08	1.855E-06	G3P2, TfR1, TGF-beta 1, GLUT1, Carbonic anhydrase XII, ALDOA, iNOS, Nucleophosmin, Adrenomedullin, PLGF, TGF-beta 3, HIF1A, PFKL, REDD1, LOXL2, MMP-2, GPI, Galectin-1, VEGF-A, 5'-NTD, ENO1, p21, LDHA, p53, MMP-9, PAI1, Carbonic anhydrase IX, PGK1, MDR1, PKM2, IBP3
10	Cell cycle_DNA replication: elongation and termination	2.393E-08	3.585E-06	Ubiquitin, PCNA, Chk1, MCM7, TOP1, MCM5, MCM4, RNASEH2C, POLD reg (p68), CDC34, Ribonuclease H1, MCM6, RFC2, E2N(UBC13), POLD reg (p50), FEN1, POLD cat (p125), LRR-1, TIPIN, UBE2D2, DCC1, CDK1 (p34), CDK2, CDC45L
11	Apoptosis and survival_TNF-alpha-induced Caspase-8 signaling	3.024E-08	4.118E-06	Acid sphingomyelinase, Bax, Caspase-7, AKT1, ErbB2, Caspase-6, PP2A structural, Caspase-3, RIPK1, PP2A regulatory, AKT2, TRADD, PP2A catalytic, AKT(PKB), HSP90, Cytochrome c, FADD, TNF-R1, Cathepsin D
12	Apoptosis and survival_p53 and p73-dependent apoptosis	3.368E-08	4.204E-06	BAD, Cystatin C, ZNHIT1, Bax, Caspase-7, GPX1, Survivin, Caspase-6, p53 (mitochondrial), PLEKHF1, E2F1, Caspase-3, DAXX, SIVA1, Prohibitin, Bcl-XL, GADD45 alpha, HtrA2, NOL3, PIG3, Pin1, AKT(PKB), Bak, p53, p38 MAPK, APEX, RanBPM, Cathepsin D
13	IGF signaling in lung cancer	4.778E-08	5.506E-06	BAD, 4E-BP1, Shc, RHEB2, Survivin, H-Ras, MAP2K5 (MEK5), MEK2(MAP2K2), MMP-2, Bcl-XL, Histone H2AX, VEGF-A, AKT(PKB), IBP4, p38 MAPK, MMP-9, IBP, PLAU (UPA), IBP3
14	Development_Regulation of epithelial-to-mesenchymal transition (EMT)	8.368E-08	8.954E-06	VE-cadherin, N-cadherin, TGF-beta 1, RelA (p65 NF-kB subunit), E2A, TGF-beta 3, WNT, SLUG, Lef-1, c-Jun, MMP-2, PDGF-A, Caldesmon, TGIF, TNF-R1, Jagged1, Vimentin, MMP-9, PAI1, PDGF-D, Frizzled, FGFR1, ACTB
15	Apoptosis and survival_BAD phosphorylation	1.242E-07	1.197E-05	BAD, Shc, Bax, Calcineurin A (catalytic), G-protein alpha-s, H-Ras, PP1-cat alpha, MEK2(MAP2K2), 14-3-3, Bcl-XL, G-protein beta/gamma, PP2C, PP2A catalytic, AKT(PKB), Cytochrome c, p70 S6 kinase2, PKA-cat (cAMP-dependent), CDK1 (p34)
16	Transcription_Negative regulation of HIF1A function	1.279E-07	1.197E-05	Ubiquitin, RACK1, FHL3, MCM7, Sirtuin3, HSC70, MCM5, Elongin B, PRDX2, HIF1A, COMMD1 (MURR1), Elongin C, HSP40, PSMA7, Sirtuin6, HSP90, Calpain 1(mu), PRDX4, CHIP, HSP70, RUVBL2, p53, SKP1, AML1 (RUNX1)
17	IGF family signaling in colorectal cancer	1.459E-07	1.286E-05	GIPC, 4E-BP1, I-kB, Shc, ZNF143, RelA (p65 NF-kB subunit), CDC25C, HIF1A, E2F1, H-Ras, c-Jun, eIF4E, GSK3 alpha/beta, Bcl-XL, MNK2(GPRK7), MEK1/2, VEGF-A, NF-kB, AKT(PKB), Rad51, IBP, IBP3
18	Development_Non-canonical TGF-beta signaling via PI3K, RhoA, and ROS	1.627E-07	1.354E-05	RhoA, 4E-BP1, TGF-beta 1, NOX4, PDIP38, TGF-beta 3, SLUG, MRLC, RhoA-related, Caldesmon, PP2A regulatory, CDC42, TGF-beta, Actin cytoskeletal, PARD6A, PP2A catalytic, AKT(PKB), HIC5, p38 MAPK, ACTB, Cofilin, Rac1, CDK2
19	Epithelial cell apoptosis in COPD	1.993E-07	1.433E-05	BAD, I-kB, PSMA1, Bax, PSMB6, KEAP1, DJ-1, Prohibitin, Bcl-XL, SP-C, PAR1, NF-kB, AKT(PKB), p53, NRF2

20	FAK1 signaling in melanoma	2.065E-07	1.433E-05	RhoA, Shc, RelA (p65 NF-kB subunit), PKC-alpha, MMP-2, MEK1/2, VEGF-A, Actin cytoskeletal, CRK, NF-kB, CAS-L, RhoC, ITGA5, MMP-9, PLAU (UPA), Paxillin, Rac1
21	FGF2 signaling in melanoma	2.065E-07	1.433E-05	STAT3, RhoA, Shc, Syndecan-1, Fra-1, MMP-2, Syndecan-4, MEK1/2, VEGF-A, CDC42, Collagen IV, p21, SPRY2, p38 MAPK, FGFR1, Rac1, CDK1 (p34)
22	Plasminogen activators signaling in pancreatic cancer	2.105E-07	1.433E-05	Shc, TGF-beta 1, RelA (p65 NF-kB subunit), Fra-1, PLAT (TPA), Annexin II, H-Ras, FOXM1, MEK2(MAP2K2), MMP-14, MMP-2, HB-EGF, Galectin-1, MMP-9, PAI1, PLAU (UPA)
23	Inhibition of remyelination in multiple sclerosis: regulation of cytoskeleton proteins	2.917E-07	1.900E-05	MLCP (cat), Tubulin beta, RhoA, CDK5, Tubulin alpha, PKC-alpha, MRLC, Tubulin beta 4, Myosin II, CDC42, TGF-beta, Actin cytoskeletal, MELC, LIMK1, Paxillin, Cofilin, Rac1, Tubulin (in microtubules)
24	Possible regulation of HSF-1/ chaperone pathway in Huntington's disease	3.255E-07	2.020E-05	HSF1, PKC-alpha, SUMO-2, HSP27, GSK3 alpha/beta, HSP40, HSP90, PLA2, HSP70, E2I, PLK1, p23 co-chaperone
25	Cell cycle_Role of APC in cell cycle regulation	3.372E-07	2.020E-05	CDC18L (CDC6), Cyclin B, CKS1, RASSF1, CDC20, MAD2b, CDH1, Tome-1, Geminin, Aurora-A, PLK1, PKA-cat (cAMP-dependent), Securin, CDK1 (p34), CDK2
26	DNA damage_p53 activation by DNA damage	4.152E-07	2.392E-05	ELAVL1 (HuR), p38alpha (MAPK14), Tip60, Chk1, Bax, DDB2, RelA (p65 NF-kB subunit), SIAH2, TTC5 (Strap), PP2A structural, E2F1, DAXX, 14-3-3, Bcl-XL, GADD45 alpha, PP2A regulatory, PIG3, PP2A catalytic, p21, p53, 14-3-3 theta, p38 MAPK, PP2C gamma
27	Signal transduction_Soluble CXCL16 signaling	4.952E-07	2.747E-05	STAT3, RhoA, I-kB, CD44, HIF1A, G-protein alpha-i family, F-Actin cytoskeleton, MMP-2, IKK-gamma, MEK1/2, G-protein beta/gamma, VEGF-A, NF-kB, AKT(PKB), p38 MAPK, MMP-9, Cofilin
28	MAPK-independent proliferation of normal and asthmatic smooth muscle cells	5.374E-07	2.870E-05	STAT3, Histamine H1 receptor, I-kB, TGF-beta 1, ErbB2, Cyclin D3, CDK4, PLAT (TPA), G-protein alpha-i family, PDGF-A, HB-EGF, PDGF-C, G-protein beta/gamma, NF-kB, AKT(PKB), G-protein alpha-q/11, p21, TBXA2R, p70 S6 kinase2, FGFR1, Rac1, IBP3
29	Role of metalloproteases and heparanase in progression of pancreatic cancer	5.557E-07	2.870E-05	Shc, CD44, CD44 soluble, H-Ras, MEK2(MAP2K2), MMP-14, MMP-2, CD44 (EXT), HB-EGF, VEGF-A, MMP-11, Collagen IV, MMP-9, CD147, TIMP1
30	Apoptosis and survival_Regulation of apoptosis by mitochondrial proteins	6.510E-07	3.250E-05	BAD, p38alpha (MAPK14), Bax, PLSCR3, Calcineurin A (catalytic), MPTP complex, SMCR7, GC1QBP, RASSF1, Fis1, SLC25A3, PP1-cat alpha, MUL1, Bcl-XL, MTCH2, HtrA2, VDAC 1, Pin1, PP2C, PP2A catalytic, PARL, VDAC 2, Bak, Calpain 1(mu), Cytochrome c, p38 MAPK, Cathepsin D, Endonuclease G, Cofilin, CDK2
31	EGFR family signaling in pancreatic cancer	7.457E-07	3.603E-05	STAT3, RhoA, I-kB, Shc, ErbB2, Survivin, CDK4, Cyclin E, H-Ras, c-Jun, MEK2(MAP2K2), MMP-2, HB-EGF, Bcl-XL, VEGF-A, NF-kB, PEA3, AKT(PKB), p21, p38 MAPK, MMP-9, PLAU (UPA), Rac1, CDK2
32	G-protein signaling_Proinsulin C-peptide signaling	9.779E-07	4.578E-05	RhoA, cPKC (conventional), PAK, ATP1A1, Calcineurin A (catalytic), iNOS, PKC-alpha, Na/K ATPase, PP1-cat, G-protein alpha-i family, MRLC, MEK1/2, G-protein beta/gamma, CDC42,

				PTP-1B, ENO1, NF-kB, AKT(PKB), LIMK, MMP-9, Histone H4, Cofilin, TIMP1, Rac1
33	ErbB2-induced breast cancer cell invasion	1.312E-06	5.954E-05	I-kB, Shc, Cofilin, non-muscle, ErbB2, PKC-alpha, H-Ras, MEK3(MAP2K3), MMP-14, MMP-2, MEK1/2, VEGF-A, CDC42, Actin cytoskeletal, NF-kB, AKT(PKB), Calpain 1(mu), ER81, p38 MAPK, MMP-9, PLAU (UPA), Cofilin, Rac1
34	Role of inhibition of WNT signaling in the progression of lung cancer	1.468E-06	6.466E-05	RhoA, N-cadherin, CD82, MAP2K5 (MEK5), c-Jun, p21, p53, Vimentin, SPRY4, Axin, MMP-9, Frizzled, TIMP1, Rac1
35	IL-6 signaling in breast cancer cells	1.532E-06	6.558E-05	STAT3, N-cadherin, Shc, RelA (p65 NF-kB subunit), MUC1, Survivin, C/EBPbeta, H-Ras, Fascin, JAB1, Bcl-XL, MEK1/2, AKT2, AKT(PKB), Jagged1, Vimentin, Carbonic anhydrase IX, MDR1, C/EBPdelta
36	HIF-1 in gastric cancer	1.914E-06	7.965E-05	Ubiquitin, PDF, GLUT1, Bax, F264, ErbB2, HIF1A, VEGF-A, CDC42, ENO1, AKT(PKB), HSP90, p21, LDHA, p53, MDR1, LAMR1, Rac1
37	CFTR folding and maturation (normal and cystic fibrosis)	2.185E-06	8.794E-05	MA1B1, OST complex, DNAJB6 (Hdj-1), GANAB, HSPBP1, HSP40, Calnexin, ERp29, Sti1, HSP70, FKBP8, p23 co-chaperone
38	Signal transduction_Non-neuronal ACM1, ACM3 and ACM5 signaling	2.231E-06	8.794E-05	MLCP (cat), RhoA, p70 S6 kinases, iNOS, G-protein alpha-s, PKC, ITGA3, PKC-alpha, G-protein alpha-12, H-Ras, MRLC, MAP2K5 (MEK5), c-Jun, G-protein alpha-11, MRLC2, G-protein alpha-q, AKT(PKB), G-protein alpha-q/11, p21, R-Ras, PKA-cat (cAMP-dependent), Rac1, CDK2
39	Tau dysregulation in Alzheimer disease	2.544E-06	9.729E-05	G3P2, p70 S6 kinases, CDK5, AKT1, Calcineurin A (catalytic), HSC70, Caspase-6, C/EBPbeta, PP1-cat, Caspase-3, PP2A regulatory, PP2A catalytic, PRNP, Calcipressin 1, AKT(PKB), APP, PPME1, PP2A cat (alpha), HSP90, Calpain 1(mu), CHIP, APP-C59 (AICD), PKA-cat (cAMP-dependent), RanBPM, Amyloid beta 42
40	Signal transduction_S1P2 receptor activation signaling	2.622E-06	9.729E-05	STAT3, MLCP (cat), RhoA, G-protein alpha-12 family, AP-1, Survivin, G-protein alpha-12, G-protein alpha-i family, H-Ras, MRLC, c-Jun, FOXM1, LIF, HB-EGF, Transgelin, MEK1/2, G-protein beta/gamma, Actin cytoskeletal, G-protein alpha-q, SMAD1, NF-kB, Osteoprotegerin, AKT(PKB), LIMK1, S1P2 receptor, p38 MAPK, G-protein alpha-i2, Paxillin, Cofilin
41	Noise-induced hair cell death and spiral ganglion neuron degeneration in deafness	2.663E-06	9.729E-05	BAD, STAT3, RhoA, p38alpha (MAPK14), Bax, AKT1, Calcineurin A (catalytic), iNOS, Histone H3, CCL13, LKB1, DIA1, AMPK beta subunit, F-Actin cytoskeleton, Caspase-3, RIPK1, AMPK gamma subunit, HDAC1, HDAC3, Isoform p66 Shc, Bak, Calpain 1(mu), Cytochrome c, p53, Actin, Endonuclease G, Rac1
42	Signal transduction_AKT(PKB) signaling	3.002E-06	1.071E-04	BAD, NT5C, GLUT1, RAG1, ACLY, AKT1, Cofilin, non-muscle, RHEB2, C/EBPbeta, Caspase-3, Connexin 43, PRAS40, GADD45 alpha, MEK1/2, AKT2, IRF3, eIF2S1, AKT(PKB), p21, ITGA5, ARPC2, Rac1
43	HCV-dependent transcription regulation leading to hepatocellular carcinoma (HCC)	3.257E-06	1.116E-04	STAT3, Bax, CDK4, Cyclin E, E2F1, Bcl-XL, p21, HSP70, p53, PKA-cat (cAMP-dependent), CDK2

44	IL-6 signaling in colorectal cancer	3.279E-06	1.116E-04	STAT3, Ku80, TGF-beta 1, Bax, Cyclin B, Survivin, Cyclin E, SOCS3, Bcl-XL, Ku70, HDAC1, Cyclin B1, HSP70, p53, CDK1 (p34)
45	DNA damage_ATM/ATR regulation of G2/M checkpoint: cytoplasmic signaling	3.730E-06	1.211E-04	MLCP (cat), p38alpha (MAPK14), Chk1, Histone H3, CDC25C, UBE2C, PP1-cat, MEK3(MAP2K3), 14-3-3, JAB1, GADD45 alpha, PP2A regulatory, PP2A catalytic, Cyclin B1, Aurora-A, PLK1, p38 MAPK, CDK1 (p34)
46	HSP70 and HSP40-dependent folding in Huntington's disease	3.783E-06	1.211E-04	Ubiquitin, PSMD1, DNAJB6 (Hdj-1), HSC70, HSP27, HSP40, HSP90, CHIP, Sti1, HSP70, SGTA, Cathepsin D
47	DNA damage_ATM activation by DNA damage	3.905E-06	1.211E-04	Tip60, p18, CDK5, BRAT1, Histone H3, OBFC2B, NK31, Histone H2B, Aven, RNF8, Histone H2AX, PP2A regulatory, TTI2, E2N(UBC13), HDAC1, PP2A catalytic, PP5, Mob2 (HCCA2), HSP90, Calpain 1(mu), Suv39H1, Histone H4
48	Signal transduction_S1P2 receptor inhibitory signaling	3.961E-06	1.211E-04	MLCP (cat), RhoA, VE-cadherin, G-protein alpha-12 family, G-protein alpha-s, G-protein alpha-12, G-protein alpha-i family, H-Ras, MRLC, MEK1/2, G-protein beta/gamma, G-protein alpha-q, Alpha-actinin, AKT(PKB), RhoC, S1P2 receptor, G-protein alpha-i2, PKA-cat (cAMP-dependent), Rac1
49	Apoptosis and survival_Caspase cascade	3.961E-06	1.211E-04	Bax, Caspase-7, Survivin, Caspase-6, RAIDD, Caspase-3, RIPK1, Caspase-4, Lamin A/C, Bcl-XL, HtrA2, VDAC 1, TRADD, Bak, Cytochrome c, FADD, TNF-R1, p53, TWEAK(TNFSF12)
50	Apoptosis and survival_NGF/ TrkA PI3K-mediated signaling	4.756E-06	1.425E-04	BAD, RhoG, MLCP (cat), RhoA, 4E-BP1, Shc, AKT1, Calcineurin A (catalytic), Cofilin, non-muscle, H-Ras, Destrin, MRLC, Myosin II, CDC42, Actin cytoskeletal, PARD6A, VAV-3, AKT(PKB), LIMK1, ARAP3, Cofilin, Rac1, Tubulin (in microtubules)
